# Elevated ITGA5 facilitates hyperactivated mTORC1-mediated progression of laryngeal squamous cell carcinoma via upregulation of EFNB2

**DOI:** 10.7150/thno.76232

**Published:** 2022-10-24

**Authors:** Dapeng Li, Anjiang Sun, Liang Zhang, Zhao Ding, Fangzheng Yi, Xue Yang, Zixi Wang, Xu Chen, Weiwei Liu, Shixian Liu, Hailong Shen, Manli Miao, Ling Zhang, Ping Liu, Yuchen Liu, Shihong Su, Hailiang Huang, Can Huang, Zhongdong Hu, Hongbing Zhang, Xiaojun Zha, Yehai Liu

**Affiliations:** 1 Department of Otorhinolaryngology, Head & Neck Surgery, The First Affiliated Hospital of Anhui Medical University, Hefei, China.; 2 Department of Biochemistry & Molecular Biology, School of Basic Medicine, Anhui Medical University, Hefei, China.; 3 State Key Laboratory of Medical Molecular Biology, Department of Physiology, Institute of Basic Medical Sciences, Chinese Academy of Medical Sciences & Peking Union Medical College, Beijing, China.; 4 Modern Research Center for Traditional Chinese Medicine, School of Chinese Materia Medica, Beijing University of Chinese Medicine, Beijing, China.

**Keywords:** mTOR, ITGA5, EFNB2, LSCC, tumorigenesis

## Abstract

**Background:** Laryngeal squamous cell carcinoma (LSCC) is one of the most common malignant tumors of the head and neck, and it has shown increasing incidence and mortality. The mechanistic target of rapamycin complex 1 (mTORC1) is frequently dysregulated in LSCC, but its underlying mechanisms remain unclear.

**Methods:** Establishment of a novel LSCC cell line using primary LSCC tumor tissues with dysregulated mTORC1 activity and then stable knockdown of Raptor (an mTORC1 specific component) in this cell line. Transcriptomic sequencing, quantitative real-time PCR, western blot analysis, and immunofluorescence assays were used to identify the crucial downstream effector of mTORC1. A series of experiments were conducted to investigate the functions and underlying mechanisms of the mTORC1 target gene in LSCC progression. Clinical LSCC samples were used to evaluate the association of mTORC1 and its downstream targets with clinicopathological features and patient prognosis. Finally, the influence on cisplatin (CDDP) sensitivity upon depletion of the mTORC1 target gene was assessed using a cell culture system, a cell line-derived xenograft (CDX) model, and a patient-derived xenograft (PDX) model.

**Results:** We successfully established a novel LSCC cell line with hyperactivated mTORC1 activity and then identified integrin subunit alpha 5 (ITGA5) as a novel functional downstream effector of mTORC1 in the progression of LSCC. Elevated ITGA5 promotes LSCC progression through augmentation of ephrin-B2 (EFNB2). Clinical data analysis indicated that the activation of the mTORC1-ITGA5-EFNB2 signaling pathway is associated with malignant progression and poor prognosis of LSCC patients. Inhibition of ITGA5 significantly sensitized LSCC cells to CDDP.

**Conclusions:** Our findings highlight a novel molecular mechanism for the tumorigenesis driven by deregulated mTORC1 signaling in LSCC, suggesting that the ITGA5-EFNB2 axis may be a therapeutic target for the treatment of mTORC1-related LSCC.

## Introduction

Laryngeal squamous cell carcinoma (LSCC) is one of the most common malignant tumors in head and neck, developing from the mucosal epithelium in the larynx, with increasing incidence and mortality 1. It is estimated that in 2020, there were 180,000 new cases and almost 100,000 deaths due to LSCC in the world 2. Tobacco consumption, alcohol abuse, and human papillomavirus (HPV) infection are the main risk factors for the development of LSCC 3. Despite advances in surgery, chemotherapy, radiotherapy, and most recently, targeted therapy and immunotherapy, a substantial number of patients still have short survival times and poor prognosis 2, 4, 5. The main reasons are that LSCC is prone to local invasion, cervical lymph node metastasis, and chemoresistance, and that there are no accurate biomarkers of therapeutic response 6. Genetic alterations and complex signaling pathways have been shown to drive continuous cancer cell survival, proliferation, and treatment resistance. Thus, there is an urgent need to elucidate the mechanism of LSCC progression and treatment resistance, so as to find effective biomarkers to predict prognosis and improve treatment strategies.

Mechanistic target of rapamycin (mTOR), also called mammalian target of rapamycin, is a highly conserved serine/threonine protein kinase that plays a critical role in a number of cellular processes, including cell growth, metabolism, autophagy, and ferroptosis, through integrating multiple inputs, such as growth factor and nutrient status 7-9. It mediates these functions primarily via interaction with several proteins to form two distinct protein complexes, mTOR complex 1 (mTORC1) and mTOR complex 2 (mTORC2). mTORC1 contains mTOR, Raptor, mLST8, PRAS40, and Deptor, whereas mTORC2 consists of mTOR, Rictor, mSIN1, mLST8, Deptor, and Protor 7, 10. While rapamycin is an effective inhibitor of mTORC1, mTORC2 is relatively resistant to rapamycin 11. Owing to mutated activation of proto-oncogenes, such as PI3K and AKT, or mutated inactivation of tumor suppressors, such as PTEN, TSC1, and TSC2, the aberrant activation of the mTORC1 signaling pathway occurs frequently in many human cancers, including LSCC, which is associated with tumorigenesis and cancer progression 12-14. Thus, rapamycin, as well as its analogues, are considered to be potential drugs against some advanced cancers 15-17. However, their therapeutic efficacy was rather limited 18, 19. The main reason of this limitation is that directly inhibition of mTORC1 by rapamycin resulted in feedback activation of AKT 20, 21. Targeting its effective downstream effectors rather than mTORC1 itself may be a productive strategy to treatment of mTORC1-related cancers. However, in LSCC, the regulatory mechanism downstream of mTORC1 remains largely obscure.

In the present study, we established a novel LSCC cell line with hyperactivated mTORC1 activity and then identified integrin subunit alpha 5 (ITGA5) as a novel downstream effector of mTORC1 in LSCC. Elevated ITGA5 promotes LSCC tumor progression through upregulation of ephrin-B2 (EFNB2). Moreover, the mTORC1-ITGA5-EFNB2 pathway is significantly activated in LSCC tissues, which correlates with malignant progression and poor prognosis of LSCC patients. In addition, inhibition of ITGA5 sensitizes LSCC cells to cisplatin (CDDP). Our findings reveal a novel molecular mechanism for tumorigenesis driven by deregulated mTORC1 signaling in LSCC, suggesting that the ITGA5-EFNB2 axis may be a therapeutic target for the treatment of mTORC1-related LSCC.

## Materials and methods

### Tumor specimens

A total of 94 LSCC and adjacent normal mucosal (ANM) tissues were acquired during routine surgeries at the First Affiliated Hospital of Anhui Medical University (Anhui, China) from 2014 to 2020. None of the patients were subjected to chemotherapy, radiotherapy, or other related antitumor therapies before surgery. The TNM staging was done referring to the American Joint Committee on Cancer (AJCC) 8th edition TNM Staging Criteria. The study was conducted in accordance with Declaration of Helsinki, and the ethical approval was obtained from the First Affiliated Hospital of Anhui Medical University Research Ethics Committee. All of the patients provided a written informed consent before participation. Detailed information of all the 94 LSCC patients is listed in Table S1-4.

### Establishment of a novel LSCC cell line (LIU-LSC-1) and cell culture

Fresh tumor tissue was isolated from a 74-year-old LSCC patient (T3N1M0) who underwent surgery, and the specimen was immediately immersed in RPMI 1640 medium (Gibco, NY, USA) containing penicillin (100 U/mL)/streptomycin (0.1 mg/mL) and amphotericin B (0.25 µg/mL) (Beyotime, Jiangsu, China). The tissue sample was washed three times in phosphate-buffered saline (PBS) and cut into small pieces. Then, small tumor masses were dissociated enzymatically in RPMI 1640 medium containing 200 U/mL type IV collagenase (Sigma, Saint Louis, MO, USA) at 37 °C for 12 h. After two rounds of washing in PBS and centrifugation, the sediments were seeded onto 60 mm Petri dishes and cultured in Epithelial Cell Complete Medium (VivaCell, Shanghai, China) with 1% penicillin/streptomycin (Beyotime). After 3 days of incubation, the cell culture medium was replaced. Cells were passaged every 3 to 4 days. Cancer-associated fibroblasts (CAFs) were removed by a brief exposure to trypsin digestion (0.25% trypsin-EDTA, Beyotime) 22. Cells were named LIU-LSC-1 and compared with the short-tandem repeat (STR) data of cell lines included in ATCC, DSMZ, JCRB and RIKEN databases. No closely matched cell lines were found (Table S5, EV < 0.8), which suggested that LIU-LSC-1 may be a new cell line. Mycoplasma analysis of this cell line was negative.

Cell source and culture conditions of murine embryonic fibroblasts (MEFs) (Tsc1^+/+^, Tsc1^-/-^, Tsc2^+/+^, and Tsc2^-/-^), HEK293T cells and LSCC cell lines (AMC-HN-8, TU177, and LIU-LSC-1) are listed in Table S6. For hypoxic exposure, cells were cultured under hypoxic (1% O_2_) or normoxic (21% O_2_) conditions for the indicated times. All cell lines were verified by STR analysis and tested for mycoplasma contamination by MycoAlert Mycoplasma Detection Kit (Lonza #LT07-118).

### Antibodies, reagents, and plasmids

All information regarding antibodies used in this study is provided in Table S7. Rapamycin (Rapa), everolimus (RAD001), deferoxamine (DFX), DAPT and MHY1485 were purchased from Selleck Chemicals (Houston, TX, USA). Jagged1-Fc was obtained from R&D system (Minneapolis, MN, USA). Lipofectamine RNAiMax was obtained from Invitrogen (Carlsbad, CA, USA). pRL-TK, pGL3-Basic, pcDNA3.0, pcDNA3.0-HA-HIF-1α, lenti-CRISPRv2 plasmids and packaging vectors (pVSVG and psPAX2) were purchased from Addgene (Cambridge, MA, USA).

### CRISPR-Cas9

The following single-guide RNAs (sgRNAs) targeting ITGA5 and TSC2 were designed by Open-access software program CRISPR and synthesized by Sangon Biotech Co., Ltd. (Shanghai, China): ITGA5-sgRNA#1, 5'-GGGGCAACAGTTCGAGCCCA-3'; ITGA5-sgRNA#2, 5'-GGAGCCACTGAGCGACCCCG-3'; TSC2-sgRNA, 5'-CACCGAACAATCGCATCCGGATGAT-3'. Oligos were cloned into the Cas9 backbone Lenti-CRISPRv2 vector. Recombinant plasmids, psPAX2 and pVSVG were co-transfected into HEK293T cells. The medium was harvested and filtered to remove cell debris 48 h later. After infection, the cells were obtained by culture over 14 days in 1.5 µg/mL puromycin (Sigma, MO, USA). Then, the cells were placed in 96-well plates and examined by microscopy the next day to be sure that only one cell was seeded per well. Clones were passaged after 10 days, and monoclonal lines were screened via western blotting for ITGA5 knockout.

### Quantitative real-time PCR (qRT-PCR) assay and RNA sequencing

The primers for qRT-PCR (provided by Sangon Biotech Co., Ltd.) are shown in Table S8. The RNA samples were sequenced on Illumina Novaseq™ 6000 (LC Sciences, Hangzhou, China).

### Chromatin immunoprecipitation (ChIP)

The ChIP assay was performed using a SimpleChIP^®^ Plus Enzymatic Chromatin IP Kit (Cell Signaling Technology, MA, USA). PCR primer sequences for the putative HIF-1α-binding region (PBR) and a nonspecific HIF-1α-binding region (NBR) of human ITGA5 were as follows: Site1, forward, 5'-CCACCCCTAATCTCCCAAATCCT-3'; reverse, 5'-TCAGGATCTTTAAGCCCAGCATTG-3'; Site2, forward, 5'-CCAAACCCGCCCAGTCTAACC-3'; reverse, 5'-GGGGGGGCATTCCTGGGT-3'; NBR, forward, 5'-CAAAGCCAGCACCAGTGAAGAGAC-3'; reverse, 5'-CCCTCCTCCCAACACACACATATATAC-3'. The primer sequences for qRT-PCR were as follows: NBR, forward, 5'-AAGCCAGCACCAGTGAAGAGAC-3'; reverse, 5'-ACTCCTGGTTCTAGCTACTTTAATCAC-3'. PBR, forward, 5'-CGCCCAGTCTAACCCAGTCCA-3'; reverse, 5'-CCTGGGTCCCTGGAACTCTGAG-3'.

### Reporter constructs and luciferase reporter assay

A 334-bp fragment of the human ITGA5 promoter (-266/+67) containing the intact HIF-1α-binding site was obtained by PCR using human genomic DNA. The primer sequences were as follows: forward, 5'-GGGGTACCTGGAAAGGAATGGGGAGGAAGGAG-3'; reverse, 5'-GAAGATCTGCGCCCGCTCTTCCCTGTCC-3'. The fragment was cloned into the *Bgl* II and *Kpn* I sites of the pGL3-Basic plasmid (ITGA5-Luc). A Q5^®^ Site-Directed Mutagenesis Kit (NEB, Ipswich, MA, USA) was used to mutate the potential HIF-1α-binding site (ITGA5-mut). The primer sequences were as follows: forward, 5'-CCCCTAAGGGAAATGGGGGGGGGGCGC-3'; reverse, 5'-TGGGGGACGCGGGCTCAG -3'. The 293T cells were then seeded into 24-well culture plates and transfected with 400 ng of ITGA5-Luc or ITGA5-mut together with 20 ng of an internal control plasmid pRL-TK and 400 ng of HA-HIF-1α-pcDNA3.0 or the empty vector pcDNA3.0. The luciferase activity was estimated using the Dual-Luciferase Reporter Assay System (Promega, USA).

### *In vitro* functional assays

Cell Counting Kit-8 (CCK-8, TargetMol, Shanghai, China), colony formation assays, wound healing and transwell assays were used to evaluate the *in vitro* functional roles of ITGA5 and EFNB2.

### Chicken chorioallantoic membrane (CAM) assay

Pathogen-free fertilized chicken eggs were purchased from Jinan SAIS Poultry Company (Shandong, China). On embryonic developmental day 8 (EDD 8), a window about 1.0 cm was opened in the shell of each egg, and sterile gelatin sponge mixed with 20 μL of cell suspension containing 2 × 10^6^ LSCC cells was planted on CAM. The CAM was separated from the eggs after fixation with stationary solution (methanol: acetone, 1:1) for 30 min on EDD 15. Then, the CAM was recorded by a digital camera, and the number of blood vessels that converged toward the implant were counted by three blind observers.

### Patient-derived xenograft (PDX) models

Freshly excised tumor tissues were obtained from LSCC patients receiving surgery at the First Affiliated Hospital of Anhui Medical University. The tissues were cut into 2 × 2 × 3-mm^3^ pieces (kept in PRMI 1640 supplemented with penicillin and streptomycin) and grafted subcutaneously into the flank of NOD/SCID mice as P1. PDX tumors were harvested and transplanted into BALB/c nude mice as P2 when the tumor reached a size of 1000 mm^3^. We followed the aforementioned protocols to transplant PDX tumor tissues into next-generation mice as P3. Twenty P3 mice were randomly assigned into four groups (five animals per group) for subsequent experiments when the tumors were detectable.

### Immunohistochemical staining (IHC) and immunofluorescence (IF) assay

IHC analysis staining was performed as previously described 23. Antibodies against ITGA5 (diluted 1:50), EFNB2 (diluted 1:200), p-S6 (diluted 1:75), Ki-67 (diluted 1:100) and CD31 (diluted 1:100) were used. A modified histologic score (H-scores, [{% of weak staining} × 1] + [{% of moderate staining} × 2] + [{% of strong staining} × 3]) was used to evaluate IHC staining 24, 25. Each staining obtained an H-score between 0 and 300, and the average of H-score for all the cases was calculated.

For IF assays, Cells were treated with DMSO, Rapa (20 nM), RAD001 (50 nM) or MHY1485 (10 µM) for 24 h and then stained as previously described 23. Primary antibodies against ITGA5 (diluted 1:50), EFNB2 (diluted 1:200), or CD44 (diluted 1:1000) and FITC-conjugated secondary antibody (diluted 1:1000) were used. DAPI (Beyotime) was used to stain nuclei. The images were captured by LSM880 + Airyscan confocal laser scanning microscope (Carl Zeiss, Oberkochen, Germany).

### Statistical analysis

All statistical analyses were performed using GraphPad Prism 6.0. Differences between two experimental groups were conducted using the two-tailed Student's *t* test. Correlations between genes were analyzed by Pearson's correlation analysis. The survival rates were calculated by the Kaplan-Meier method. The receiver operating characteristic (ROC) curves were used to evaluate the sensitivity and specificity of genes as diagnostic biomarkers. P < 0.05 was considered statistically significant.

More detailed methods are provided in Supplemental Methods.

## Results

### Establishment and characterization of a novel LSCC cell line, LIU-LSC-1, with hyperactivated mTORC1 activity

**Figure 1:**
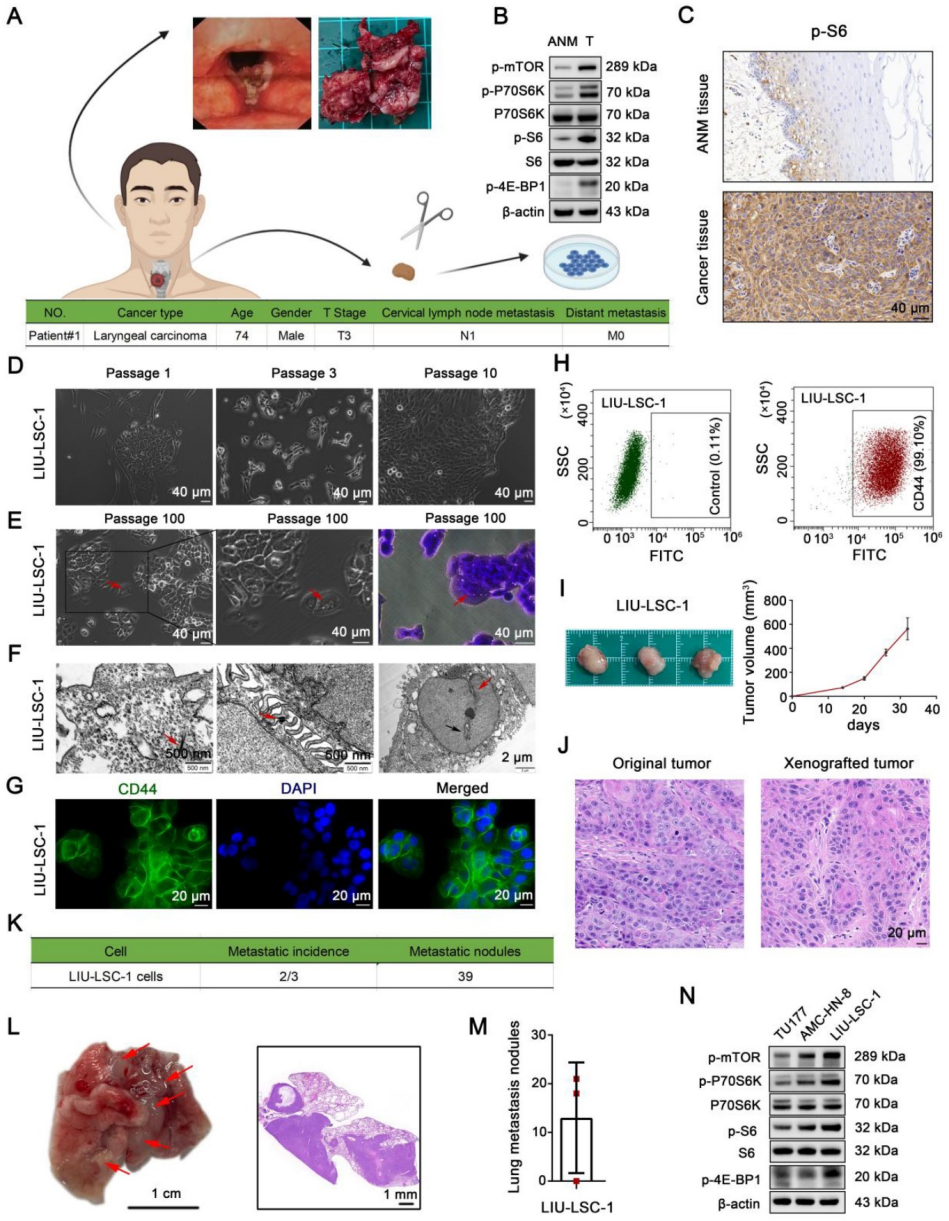
** Establishment and characterization of a novel LSCC cell line, LIU-LSC-1. (A)** Flowchart of a patient-derived cell line establishment (upper panel) and clinical characteristics of the donor (lower panel). **(B-C)** Tumor and ANM tissues were obtained from the donor during surgical resection. The level of mTORC1 activity (indicated by p-P70S6K, p-S6 and p-4E-BP1) was detected using western blotting **(B)** or IHC staining **(C)**. **(D)** Representative images of the early LIU-LSC-1 cells. **(E)** LIU-LSC-1 cells with typical malignant cell morphology, including enlarged and misshapen nuclei, cytoplasmic vacuoles, occasional multinucleated giant cells (indicated by the red arrow), and rounded and luminescent cells. LIU-LSC-1 cells were stained using crystal violet (right panel). **(C-E)** Scale bar, 40 µm. **(F)** Ultrastructural features (indicated by arrows) of LIU-LSC-1 cells were observed by transmission electron microscopy. Representative images of tonofilaments in the cytoplasm (left panel, scale bar, 500 nm), desmosomes in the intercellular connections (middle panel, scale bar, 500 nm), intranuclear inclusions (right panel, indicated by black arrow) and indented nuclear membrane (right panel, indicated by red arrow, scale bar, 2 µm) in LIU-LSC-1 cells. **(G)** IF staining of CD44 in LIU-LSC-1 cells. Scale bar, 20 µm. **(H)** Flow cytometry analysis. The negative control was stained with an isotype control antibody (left panel) and CD44 was used as a positive marker (right panel).** (I-J)** The LIU-LSC-1 cells were inoculated subcutaneously into nude mice. Tumor images and tumor volume **(I)**, representative H&E images staining from original and xenografted tumor** (J)**. **(K-M)** LIU-LSC-1 cells were injected into nude mice via the tail vein (n = 3 mice). Statistical results **(K)**. Representative images of lung (Scale bar, 1 cm) and H&E-stained lung sections (Scale bar, 1 mm), metastatic lung nodules are indicated by red arrows **(L)**. Numbers of lung metastasis nodules from each mouse **(M)**. **(N)** The indicated LSCC cells were subjected to immunoblotting with p-mTOR, p-P70S6K, p-4E-BP1 and p-S6 antibodies.

We successfully established a new LSCC cell line, designated LIU-LSC-1, using a fresh sterile specimen derived from the primary tumor of a LSCC patient (Figure 1A). As compared to the ANM tissues, mTORC1 (phospho-P70S6K^Thr389^, phospho-4E-BP1^Thr37/46^ and phospho-S6^Ser235/236^ as indicators of mTORC1 activity) is aberrantly activated in the donor tumor tissues (Figure 1B-C). LIU-LSC-1 cells grew as a cobblestone monolayer and showed typical malignant cell morphology (Figure 1D-E). To date, the primary culture cells have been successfully subcultured for more than 100 generations without showing changes in morphology. Electron microscopy revealed that LIU-LSC-1 cells have typical epithelial morphologic characteristics, such as bundles of tonofilaments in the cytoplasm, numerous desmosomes in the intercellular connections, intranuclear inclusions, and irregular nuclei contours with deep indentation of the nuclear membranes (Figure 1F). The high-level expression of CD44, a cancer stem cell marker, was confirmed by fluorescence microscopy in LIU-LSC-1 cells (Figure 1G). The purity of LIU-LSC-1 cells was about 99.10% as determined by flow cytometry (Figure 1H). The STR profile did not match any cell lines deposited in public cell banks, proving that LIU-LSC-1 is a new cell line (Table S5). The tumorigenic and metastatic properties of LIU-LSC-1 cells were assessed using nude mice. As shown in Figure 1I, subcutaneous tumors developed in all three mice. HE staining indicated that the xenografted tumors show histological characteristics of middle-differentiated squamous cell carcinoma, consistent with the original tumors (Figure 1J). Lung metastatic nodules were detected in two of the three mice upon tail vein injection of LIU-LSC-1 cells (Figure 1K-M). In addition, similar to the original tumors, LIU-LSC-1 cells showed high levels of mTORC1 activity, significantly higher than those of two commercially available LSCC cell lines: TU177 and AMC-HN-8 cells (Figure 1N). Collectively, we established a highly tumorigenic LSCC cell line with dysregulated activity of mTORC1.

### mTORC1 positively regulates ITGA5 expression in LSCC

**Figure 2:**
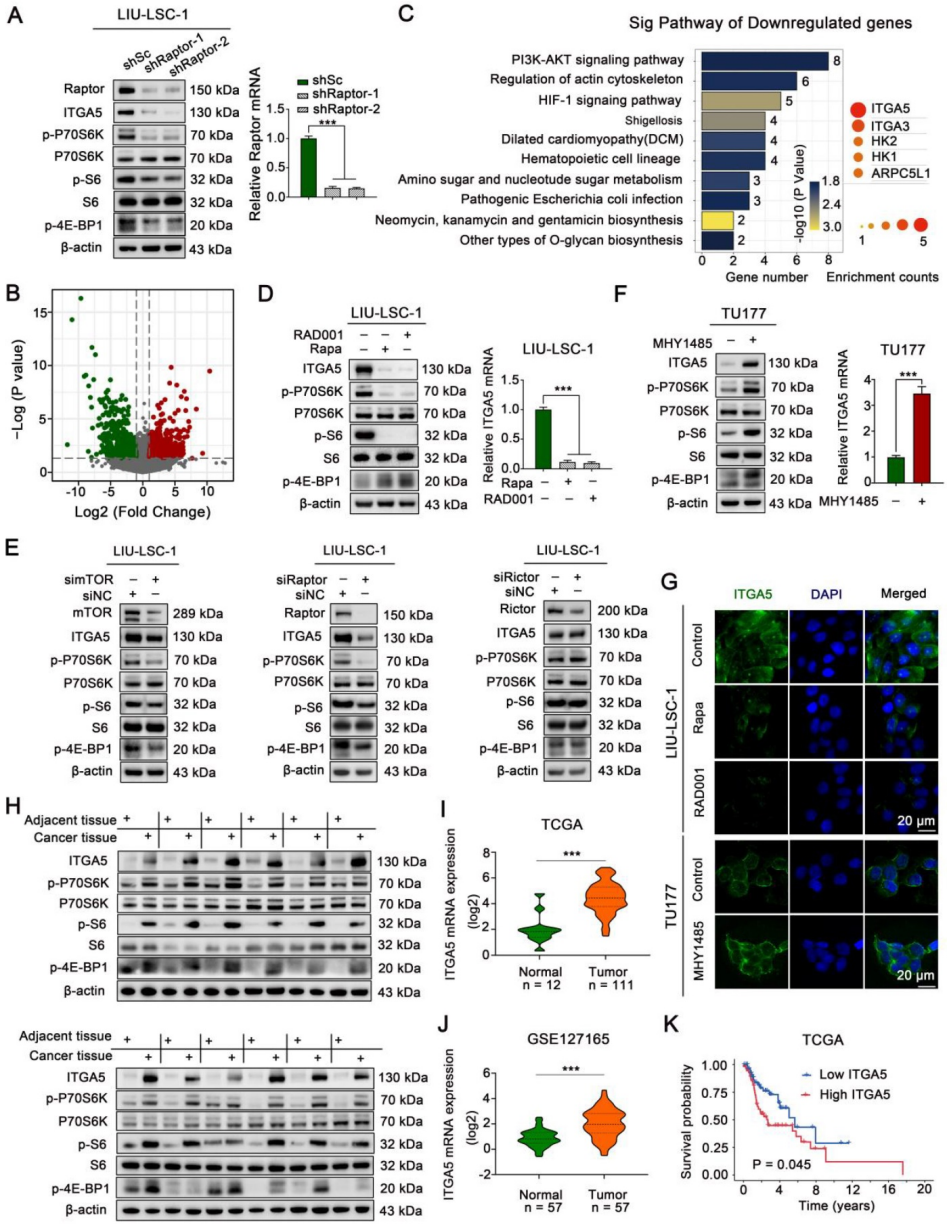
** mTORC1 positively regulates ITGA5 expression in LSCC. (A)** LIU-LSC-1 cells were stably expressing shRNAs targeting Raptor (shRaptor) or a control shRNA (shSc), western blotting and qRT-PCR were used to measure protein and mRNA levels. **(B-C)** RNA-sequencing analysis of shRaptor-1 LIU-LSC-1 cells and the controls cells (shSc LIU-LSC-1 cells) identified 224 upregulated and 221 downregulated DEGs, respectively. Volcano plot **(B)** of downregulated (green) and upregulated DEGs (red). KEGG pathway analysis showing top 10 enriched pathways of downregulated DEGs **(left panel of C)** and five genes that appeared more than twice in these pathways **(right panel of C)**. The size of the circle represents the frequency of occurrence. **(D)** LIU-LSC-1 cells were treated with 20 nM Rapa or 50 nM RAD001 for 24 h.** (E)** LIU-LSC-1 cells were transfected with siRNA targeting mTOR (simTOR), Raptor (siRaptor), Rictor (siRictor) or the control (siNC) for 48 h. **(F)** TU177 cells were treated with 10µM MHY1485 for 24 h. **(D-F)** The samples were subjected to western blotting **(E, left panels of D and F)** and qRT-PCR **(right panels of D and F)** analyses.** (G)** The treated cells were subjected to IF assay. Scale bars, 20 µm. **(H)** ITGA5, p-P70S6K, p-4E-BP1 and p-S6 protein levels were measured by western blot using the paired ANM tissues and LSCC tissues (n = 12). **(I-J)** Expression analysis of ITGA5 in LSCC and normal tissues using TCGA** (I)** and GSE127165 **(J)**. **(K)** Kaplan-Meier plot for overall survival of LSCC patients in TCGA dataset based on ITGA5 expression. High (n = 55) and low (n = 56) ITGA5 expression were stratified by the median expression level. The error bars represent the mean ± SD of triplicate technical replicates. ***P < 0.001.

To explore the underlying mechanisms by which activated mTORC1 promotes tumorigenesis in LSCC, the mTORC1 specific component Raptor was stably knocked down in LIU-LSC-1 cells (Figure 2A). Compared with that of the control cells (shSc LIU-LSC-1 cells), RNA-sequencing analysis identified a total of 445 differentially expressed genes (DEGs, 224 upregulated genes and 221 downregulated genes) in shRaptor-1 LIU-LSC-1 cells (Figure 2B and Table S9). KEGG enrichment analysis for the DEGs revealed that ITGA5 was the most frequently enriched gene in the top 10 downregulated pathways, suggesting ITGA5 as a critical downstream effector of mTORC1 in LSCC (Figure 2C and Table S10).

The remarkably decreased expression of ITGA5 in Raptor stable knockdown LIU-LSC-1 cells as compared to the control cells was confirmed by western blot analysis (Figure 2A). Pharmacologic inhibition of mTORC1 with Rapa or RAD001 also led to a significant reduction in ITGA5 expression at both mRNA and protein levels in LIU-LSC-1 cells (Figure 2D). Moreover, knockdown of mTOR or Raptor significantly suppressed the expression of ITGA5, whereas knockdown of the mTORC2 specific component Rictor had little effect on ITGA5 expression, further confirming the role of mTORC1 in controlling the expression of ITGA5 in LSCC cells (Figure 2E). In contrast, activation of mTORC1 either by treatment with MHY1485 or by knockout of TSC2 led to upregulation of ITGA5 in TU177 cells (Figure 2F and Figure S1A-B). The membrane localization and mTORC1-dependent expression pattern of ITGA5 were further corroborated by immunofluorescence analysis (Figure 2G). Furthermore, western blot analysis revealed that ITGA5, along with p-P70S6K, p-S6, p-4E-BP1, was markedly upregulated in 12 paired human LSCC lesions relative to the corresponding ANM tissues (Figure 2H). Moreover, the analysis of publicly available datasets The Cancer Genome Atlas (TCGA) and GSE127165 showed that ITGA5 was remarkably increased in LSCC tissues compared with ANM tissues (Figure 2I-J), and negatively correlated with overall survival (OS) in LSCC patients (Figure 2K). Taken together, these data indicate that ITGA5 is a downstream target of mTORC1 and it may play a critical role in hyperactivated mTORC1-mediated LSCC progression.

HIF-1α is a well-known target of mTORC1 26. Inhibition of mTORC1 by rapamycin or RAD001 treatment resulted in significantly reduced expression of HIF-1α in LIU-LSC-1 and TSC2 KO1 TU177 cells (Figure S2A). In addition, there is a significant correlation between ITGA5 and HIF-1α in the LSCC datasets from TCGA and GSE127165 (Figure S2B). Therefore, HIF-1α may be a candidate transcription factor involved in mTORC1-mediated upregulation of ITGA5. We inhibited the expression of HIF-1α by transfecting specific shRNAs against HIF-1α into LIU-LSC-1 and TSC2 KO1 TU177 cells. Not surprisingly, knockdown of HIF-1α led to marked downregulation of ITGA5 (Figure S2C-D). In addition, silencing of HIF-1α obviously suppressed hypoxia-induced (DFX or 1% O_2_) ITGA5 upregulation in LIU-LSC-1 or TSC2 KO1 TU177 cells (Figure S2E-F).

To further elucidate the underlying mechanisms by which HIF-1α regulates ITGA5, the human *ITGA5* gene was examined for matches to the consensus HIF-1α binding site (Hypoxia response element, HRE) 5'-A/GCGTG-3'. Two potential HRE (site 1, 5'-ACGTG-3', -1890 to -1886; site 2, 3'-GTGCG-5', -129 to -125) was identified (Figure S2G), and the recruitment of HIF-1α on site 2 in LIU-LSC-1 cells was confirmed by ChIP assays (Figure S2H). Next, we cloned a 334-bp fragment encompassing this HRE (-266/+67) into pGL3-Basic plasmid for evaluation of promoter activity. The reporter assay suggested that HIF-1α significantly enhanced the luciferase activity of the reporter, while the enhanced transcriptional activity was markedly attenuated when the binding site was mutated (Figure S2I). Furthermore, real-time PCR analysis of ChIP DNA revealed that the enhanced integration of HIF-1α with this binding element induced by hypoxia was abolished by treatment with rapamycin in LIU-LSC-1 cells (Figure S2J). Taken together, HIF1-α promotes ITGA5 transcription by direct binding to the HRE of the *ITGA5* gene.

### ITGA5 promotes LSCC tumor progression *in vitro* and *in vivo*

**Figure 3:**
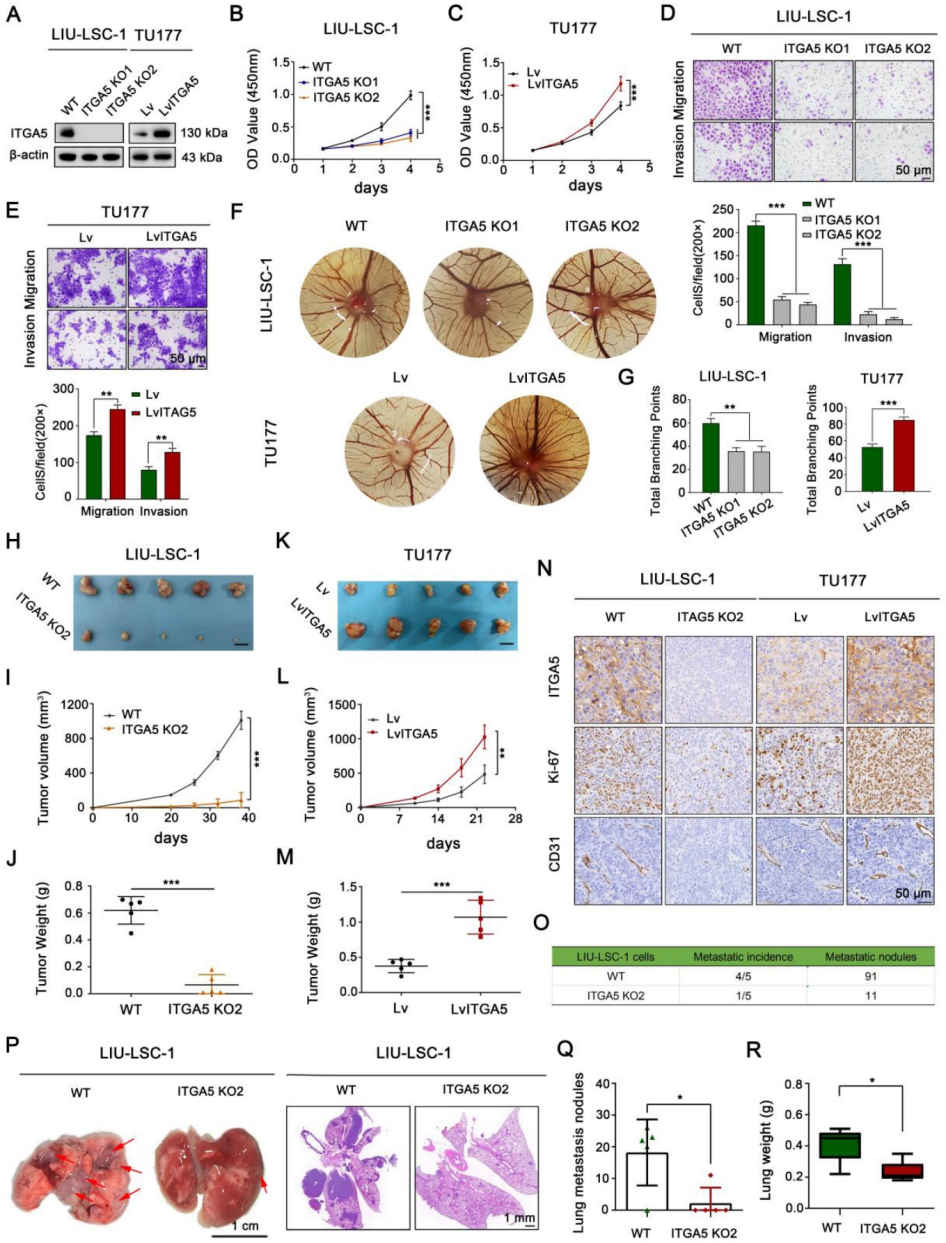
** The tumor-promoting effects of ITGA5 in LSCC cells. (A)** ITGA5 knockout LIU-LSC-1 cell lines were constructed using a CRISPR/Cas9 approach **(left panel)**. TU177 cells were infected with control lentiviruses or lentiviruses expressing ITGA5 **(right panel)**. The expression of ITGA5 was assessed by western blot.** (B-C)** CCK-8 assays were performed to evaluate cell growth rates of the indicated cells. **(D-E)** Cell migration and invasion abilities of the indicated cells were measured by transwell assays (**upper panel:** representative images; **lower panel:** statistical analysis). **(B-E)** The error bars represent the mean ± SD of triplicate technical replicates. **P < 0.01; ***P < 0.001.** (F-G)** On EDD 8, sterile gelatin sponge mixed with the indicated cells suspension was planted on chicken chorioallantoic membrane. Representative images **(F)** and statistical analysis **(G)** on EDD 15 are shown. Error bars represent mean ± SD (n = 6 per group). **P < 0.01; ***P < 0.001. **(H-N)** The indicated cells were inoculated subcutaneously into nude mice, following by monitoring for tumor growth. Tumor images **(H, K)**, tumor volumes at the indicated times **(I, L)**, tumor weight **(J, M)**, and representative IHC images of ITGA5, Ki-67, and CD31 staining from subcutaneous xenograft tissues** (N)**. Scale bars, 1 cm** (H, K)**, 50 µm **(N)**. **(O-R)** The indicated LIU-LSC-1 cells were injected into nude mice via the tail vein (n = 5 mice/group). The number of metastatic lung nodules was summarized **(O)**. Representative images of the lung (**left panel of P**, scale bar, 1 cm) and H&E-stained lung sections (**right panel of P**, scale bar, 1 mm) showing metastatic lesions generated from the indicated cells after tail vein injections. The red arrowheads point to the metastatic lung nodules. The lung metastatic nodules **(Q)** and lung weights **(R)** of all animals were summarized. Error bars indicate mean ± SD (n = 5 mice/group). *P < 0.05.

We established a stable ITGA5 knockout (KO) LIU-LSC-1 cell line to clarify the biological functions of ITGA5 in LSCC. In addition, ITGA5 was ectopically overexpressed in TU177 cells with relatively low expression of ITGA5. The efficacy of ITGA5 knockout and overexpression in these constructed cell lines was verified by western blot (Figure 3A). CCK-8 and colony formation assays showed that knockout of ITGA5 significantly reduced cell viability and colony formation ability in LIU-LSC-1 cells (Figure 3B and Figure S3A). In contrast, ITGA5 overexpression (OE) promoted cell proliferation and colony formation in TU177 cells (Figure 3C and Figure S3B). Next, we examined whether ITGA5 contributed to motility of LSCC cells. Wound healing and transwell assays demonstrated that cell migration and invasion markedly increased after ITGA5 overexpression and decreased after ITGA5 knockout (Figure S3C-D and Figure 3D-E). Furthermore, the angiogenesis activities of ITGA5 were evaluated by a CAM assay. As displayed in Figure 3F-G, ITGA5 KO LIU-LSC-1 cells induced a lower angiogenic response than the control cells, while ITGA5-overexpressing TU177 cells facilitated the formation of blood vessels toward the graft compared with the control cells.

To further verify the tumor-promoting role of ITGA5 in LSCC, ITGA5 KO2 LIU-LSC-1 cells, ITGA5 OE TU177 cells, and their corresponding control cells were subcutaneously injected into the right armpits of nude mice. As shown in Figure 3H-J, depletion of ITGA5 significantly decreased the tumorigenic capacity of LIU-LSC-1 cells compared with the controls. In contrast, the tumor sizes and weights of the ITGA5 OE group were obviously greater than those in the vector control group (Figure 3K-M). Furthermore, IHC analysis showed that staining levels of Ki-67 (one of the key indicators of cell growth) and CD31 (an angiogenic marker) were significantly decreased in tumor tissues with deletion of ITGA5, but were more intense in ITGA5-overexpressing tumor tissues (Figure 3N). In addition, we assessed the effects of ITGA5 on lung metastasis, observing that the number and the volume of metastatic nodules were significantly decreased in the ITGA5 knockout group (Figure 3O-Q). Consistently, the wet lung weight of the ITGA5 KO2 LIU-LSC-1 cells beard mice was much lighter than that of the control group (Figure 3R).

To further confirm the vital role of ITGA5 in the mTORC1-activated LSCC cells, we knocked down ITGA5 in TSC2 KO1 TU177 cells (Figure S4A). As shown in Figure S4B-J, knockout of TSC2 promoted the proliferation, migration, invasion, angiogenesis, and tumor growth abilities of TU177 cells, while these properties were significantly attenuated after depletion of ITGA5. In summary, ITGA5, as a crucial downstream effector of mTORC1, promotes cell proliferation, metastasis, angiogenesis, and tumor growth of LSCC.

### EFNB2 is a downstream target of the mTORC1-ITGA5 signaling pathway

**Figure 4:**
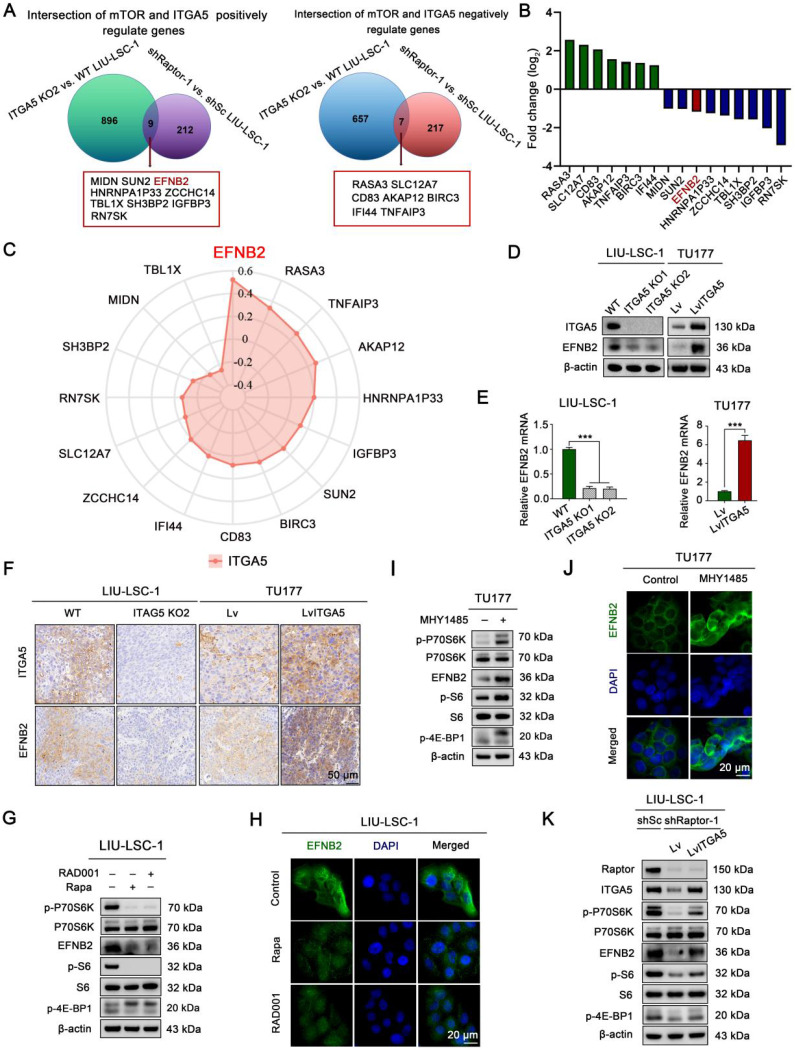
** EFNB2 is a downstream target of the mTORC1-ITGA5 signaling pathway. (A)** Schematic illustration for screening of common differential genes (co-DEGs) of the two RNA-sequencing data (ITGA5 KO2 LIU-LSC-1 cells vs. WT LIU-LSC-1 cells and shRaptor-1 LIU-LSC-1 cells vs. shSc LIU-LSC-1 cells). **(B)** mRNA expression levels of the co-DEGs in the RNA sequencing (ITGA5 KO2 LIU-LSC-1 cells vs. WT LIU-LSC-1 cells).** (C)** Radar plots were used to demonstrate Pearson's correlations between the expression levels of the co-DEGs and ITGA5 based on TCGA datasets of LSCC. **(D-E)** EFNB2 expression in the indicated cells was detected by immunoblotting** (D)** and qRT-PCR** (E)**. Error bars represent the mean ± SD of triplicate technical replicates. ***P < 0.001. **(F)** Representative IHC images of ITGA5 and EFNB2 staining from the indicated tumor tissues. Scale bars, 50 µm. **(G-H)** LIU-LSC-1 cells were treated with 20 nM Rapa or 50 nM RAD001 for 24 h. **(I-J)** TU177 cells were treated with 10 µM MHY1485 for 24 h. **(G-J)** The samples were detected by western blot analysis **(G, I)** and IF assay **(H, J)**, scale bars, 20 µm. **(K)** LIU-LSC-1 cells which endogenous Raptor was abolished by shRNA were infected with lentivirus harboring a vector encoding human ITGA5 or the empty vector. The cell lysates were subjected to western blot analysis.

To explore downstream target genes of the mTORC1-ITGA5 pathway, we first analyzed differences in gene expression profiles between ITGA5 KO2 LIU-LSC-1 cells and the wild type (WT) cells using RNA sequencing. Relative to the WT cells, 1569 DEGs (664 upregulated genes and 905 downregulated genes) in ITGA5 KO2 cells were identified (Table S11). Then, we intersected these data with the RNA-sequencing data of shRaptor-1 LIU-LSC-1 cells and the control cells. A total of 16 genes (nine positively and seven negatively regulated by the mTORC1-ITGA5 pathway) were screened (Figure 4A-B). To further narrow the potential target genes, the TCGA database was used to evaluate the association between these genes and ITGA5 in LSCC. As shown in Figure 4C, EFNB2 showed the highest correlation with ITGA5 in LSCC. Thus, we considered EFNB2 a promising potential effector of the mTORC1-ITGA5 signaling pathway, and hence, focused on it in the following experiments.

To further confirm the relationship between ITGA5 and EFNB2, we detected the expression of EFNB2 in ITGA5 knockout or overexpression LSCC cells. As showcased in Figure 4D-E, knockout ITGA5 led to downregulation of EFNB2 in LIU-LSC-1 cells, while overexpression of ITGA5 resulted in upregulated expression of EFNB2 in TU177 cells. IHC staining also showed that tumor sections from nude mice that had been injected subcutaneously with ITGA5 KO2 LIU-LSC-1 cells expressed less EFNB2 protein, while ITGA5 OE TU177 cells showed a higher expression level of EFNB2 (Figure 4F). Therefore, EFNB2 is a downstream target of ITGA5 in LSCC.

To verify that EFNB2 expression is controlled by mTORC1, we examined the expression level of EFNB2 in LSCC cells treated with mTORC1 inhibitors. As shown in Figure 4G-H, inhibition of mTORC1 by Rapa or RAD001 led to significant downregulation of EFNB2 in LIU-LSC-1 cells. To the contrary, activation of mTORC1 resulted in marked upregulation of EFNB2 in TU177 cells (Figure 4I-J). Moreover, ectopic expression of ITGA5 largely rescued EFNB2 levels in Raptor-knockdown LIU-LSC-1 cells (Figure 4K). In summary, the mTORC1-ITGA5 pathway positively regulates the expression of EFNB2 in LSCC cells.

### EFNB2 exhibits the tumor-promoting functional characteristics similar to ITGA5

**Figure 5:**
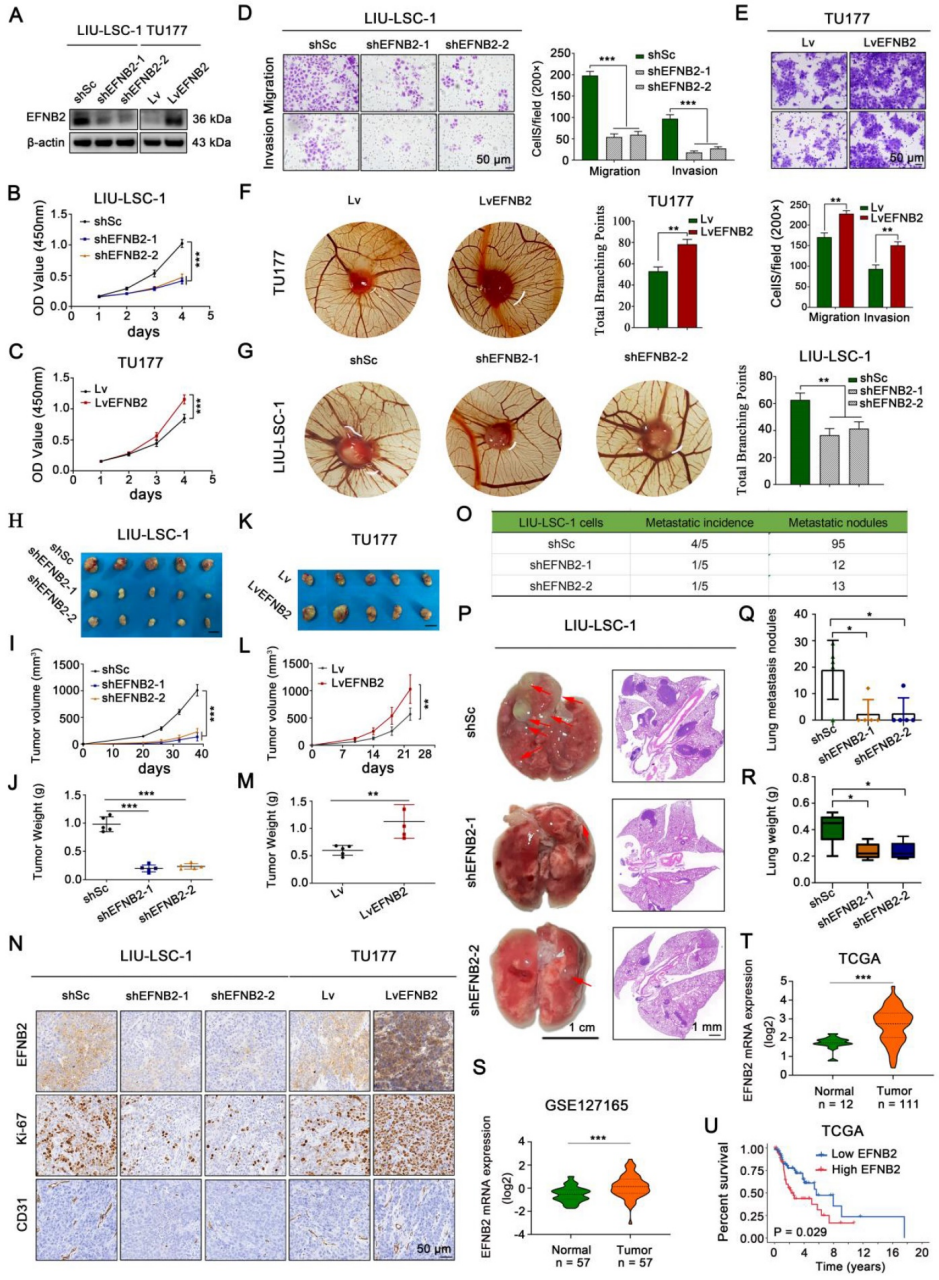
** EFNB2 exhibits tumor promoter activities in LSCC cells. (A-G)** LIU-LSC-1 cells were transduced with EFNB2 shRNAs-expressing (shEFNB2-1 or shEFNB2-2) lentiviruses or shSc; TU177 cells were infected with lentiviruses expressing either EFNB2 or empty control vector. EFNB2 expression was assessed by western blotting **(A)**. The indicated cells were subjected to CCK-8 assays** (B-C)**, transwell assays **(D-E)**, and CAM assays **(F-G)**. **(B-E)** The error bars represent the mean ± SD of triplicate technical replicates. **P < 0.01; ***P < 0.001. Scale bars, 50 µm. **(F-G)** Error bars represent mean ± SD (n = 6 per group). **P < 0.01. **(H-N)** The indicated LSCC cells were subcutaneously injected into mice for monitoring tumor growth. Tumor images** (H, K)**, tumor volume **(I, L)**, tumor weight **(J, M)**, and representative IHC images of EFNB2, Ki-67, and CD31 (N) were shown. Scale bars, 1 cm **(H, K)**, 50 µm **(N)**. Error bars, mean ± SD (n = 5 mice/group). **P < 0.01; ***P < 0.001. **(O-R)** Nude mice were injected with the indicated LIU-LSC-1 cells via the tail vein (n = 5 mice/group). The number of metastatic lung nodules was quantified **(O)**. Representative images of the lung (**left panel of P**, scale bar, 1 cm) and H&E-stained lung sections (**right panel of P**, scale bar, 1 mm) are presented. The red arrowheads point to the metastatic lung nodules. The lung metastatic nodules** (Q)** and lung weights **(R)** of all animals were summarized. Error bars indicate mean ± SD (n = 5 mice/group). *P < 0.05. **(S-T)** Expression analysis of EFNB2 in LSCC tissues and normal tissues using GSE127165 **(S)** and TCGA **(T)** databases. **(U)** Kaplan-Meier plot for overall survival of LSCC patients in TCGA dataset based on EFNB2 expression. High (n = 55) and low (n = 56) EFNB2 expression were stratified by the median expression level.

To explore the pro-oncogenic role of EFNB2 in LSCC, two shRNAs specially targeting EFNB2 were transfected into LIU-LSC-1 cells, and we also engineered TU177 cells to stably overexpress EFNB2. The knockdown and overexpression efficiencies in the transformed cell lines were detected by western blot (Figure 5A). By CCK-8 and colony formation assays, we confirmed that knockdown of EFNB2 reduced the proliferative capacity of LIU-LSC-1 cells (Figure 5B and Figure S5A). Moreover, wound healing assays and transwell assays revealed that the migration and invasion abilities of LIU-LSC-1 cells were significantly inhibited through the downregulation of EFNB2 expression level (Figure 5D and Figure S5C). Additionally, knockdown of EFNB2 inhibited the angiogenic ability of LIU-LSC-1 cells in a CAM assay (Figure 5G). In line with LIU-LSC-1 cells, knockdown of EFNB2 led to similar results in TSC2 KO1 TU177 cells (Figure S6A-F). In contrast, overexpression of EFNB2 promoted the proliferation, migration, invasion, and angiogenesis of TU177 cells (Figure 5C, Figure 5E-F, Figure S5B and Figure S5D).

To further define the functional role of EFNB2 in regulation of LSCC progression *in vivo*, we performed the xenograft tumor formation assay. Consistent with the results *in vitro*, EFNB2 knockdown markedly retarded tumor progression, whereas EFNB2 overexpression rigorously promoted tumor growth (Figure 5H-M and Figure S6G-I). IHC analysis of Ki-67 and CD31 in the xenograft tumors further confirmed that EFNB2 was a positive regulator of cell proliferation and angiogenesis *in vivo* (Figure 5N). Moreover, we evaluated the EFNB2 affection in metastasis using the tail vein injection model. Eight weeks after injection, we found that, similar to the knockout of ITGA5, the nude mice in the EFNB2 knockdown groups produced fewer lung metastases nodules compared with the control group (Figure 5O-R). In addition, we evaluated the clinical significance of EFNB2 in LSCC by using public datasets TCGA and GSE127165. EFNB2, in line with ITGA5, was highly expressed in cancer tissues, compared with normal tissues (Figure 5S-T), and high expression of EFNB2 conferred a poor prognosis in LSCC patients (Figure 5U). To summarize, in line with ITGA5, EFNB2 plays a role of an oncogene in LSCC cells.

### ITGA5 promotes proliferation, angiogenesis, and metastasis of LSCC cells through upregulation of EFNB2

**Figure 6:**
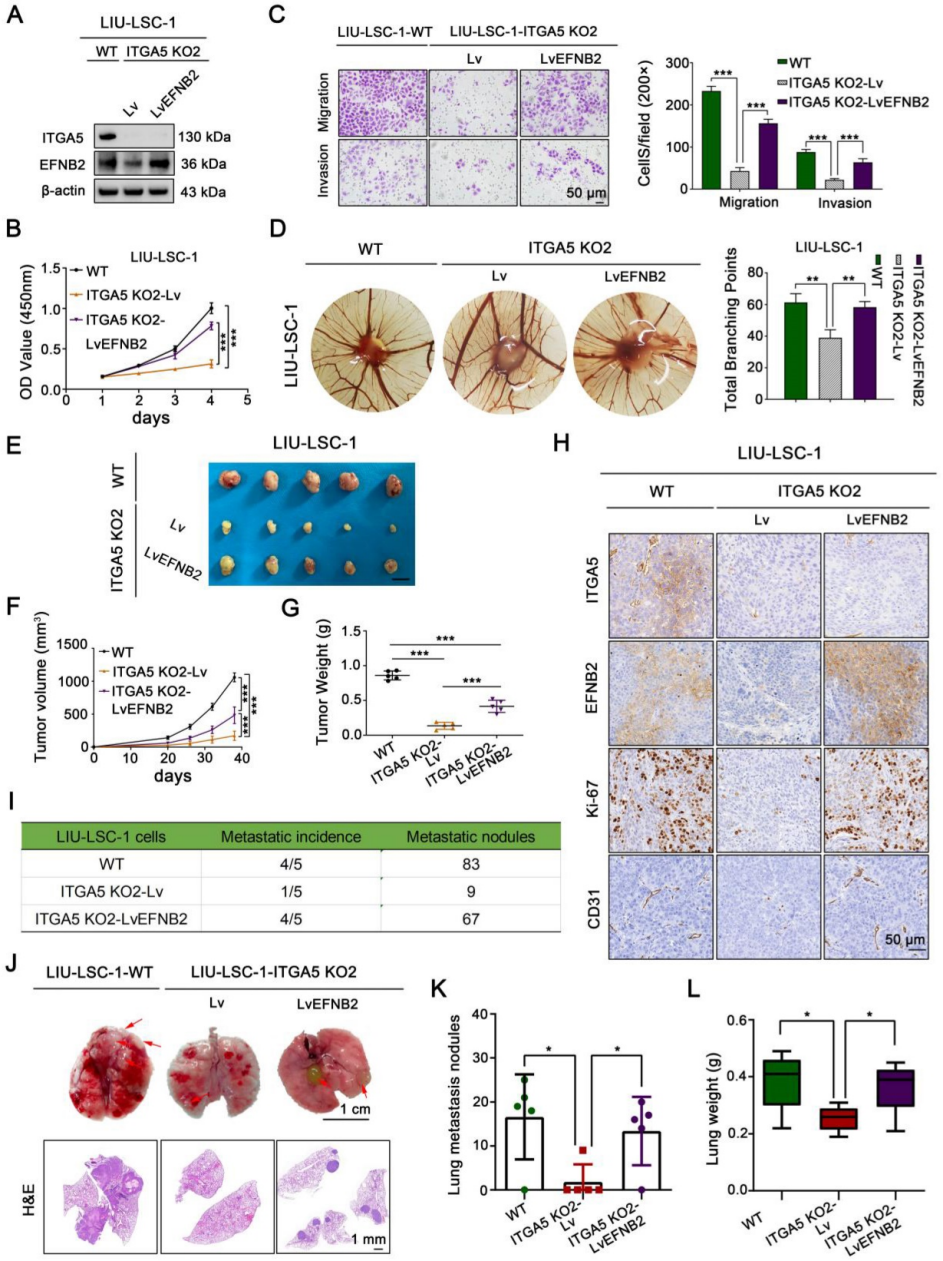
** EFNB2 is a functionally important downstream target of ITGA5 in LSCC. (A)** ITGA5 KO2 LIU-LSC-1 cells were infected with lentiviruses harboring a vector encoding human EFNB2 or the empty vector. The cell lysates were subjected to immunoblotting.** (B-C)** Cell growth rates, migration and invasion abilities of the indicated cells were evaluated by CCK-8 assays **(B)** and transwell assays (**C, left panel**: representative images; **right panel**: statistical analysis). Scale bars, 50 µm. Data were indicated as mean ± SD of triplicate technical replicates. ***P < 0.001. **(D)** The indicated cells were subjected to CAM assays. Representative images **(left panel)** and statistical analysis **(right panel)** are shown. Error bars represent mean ± SD (n = 6 per group). **P < 0.01. **(E-H)** Nude mice received subcutaneous injections with the indicated LSCC cells. Tumor images **(E)**, tumor volume **(F)**, tumor weight **(G)**, and representative IHC images **(H)** of xenograft tumors inoculated by the indicated cells. Error bars, mean ± SD (n = 5 mice/group). ***P < 0.001. Scale bars, 1 cm **(E)**, 50 µm **(H)**. **(I-L)** The indicated LIU-LSC-1 cells were injected into nude mice via the tail vein (n = 5 mice/group). The number of metastatic lung nodules was summarized **(I)**. Representative images of lung (Scale bar, 1 cm) and H&E-stained lung sections (Scale bar, 1 mm) **(J)** showing metastatic lesions generated from the indicated cells after tail vein injections. Red arrowheads point to metastatic lung nodules. Lung metastatic nodules **(K)** and lung weight** (L)** of all animals were summarized. Error bars, mean ± SD (n = 5 mice/group). *P < 0.05.

To investigate whether the pro-oncogenic effect of ITGA5 is dependent on EFNB2, we first overexpressed EFNB2 in ITGA5 KO2 LIU-LSC-1 cells (Figure 6A). As shown in Figure 6B-C and Figure S7A-B, restoration of EFNB2 efficiently reversed the adverse effects of ITGA5 knockout on LIU-LSC-1 cell proliferation, migration, and invasion. Meanwhile, EFNB2 overexpression weakened the inhibitory effect of ITGA5 loss on angiogenesis (Figure 6D). We also transduced a lentiviral vector expressing shRNA for EFNB2 to ITGA5-overexpressing TU177 cells (Figure S8A). As expected, the accelerated cell proliferation, migration, invasion, and angiogenesis driven by ITGA5 overexpression were abolished by knockdown of EFNB2 in TU177 cells (Figure S8B-F). Taken together, these data underscore the importance of ITGA5-regulated EFNB2 expression in the tumorigenesis of LSCC.

The mediation effect of EFNB2 on the ITGA5-related regulation of LSCC growth and metastasis was further verified *in vivo* using a subcutaneous xenograft tumor model and a tail vein lung cancer metastasis mouse model. Consistent with the *in vitro* results, EFNB2 re-expression abrogated the suppressive effects of ITGA5 depletion on xenograft growth, tumor cell proliferation (as assessed by Ki-67 index), and tumor angiogenesis (as assessed by CD31 index) (Figure 6E-H). The reduced number and size of pulmonary metastatic nodules in animal groups injected with ITGA5 KO2 LIU-LSC-1 cells were also partially rescued by overexpression of EFNB2, as confirmed by macroscopic observation, hematoxylin and eosin (H&E) staining of pulmonary tissues, and scaling of the average lung weight of tumor-bearing mice (Figure 6I-L). Collectively, ITGA5 promotes LSCC growth and metastasis through enhancing EFNB2 expression.

### Activation of the mTORC1-ITGA5-EFNB2 signaling pathway is associated with malignant progression and poor prognosis of LSCC

**Figure 7:**
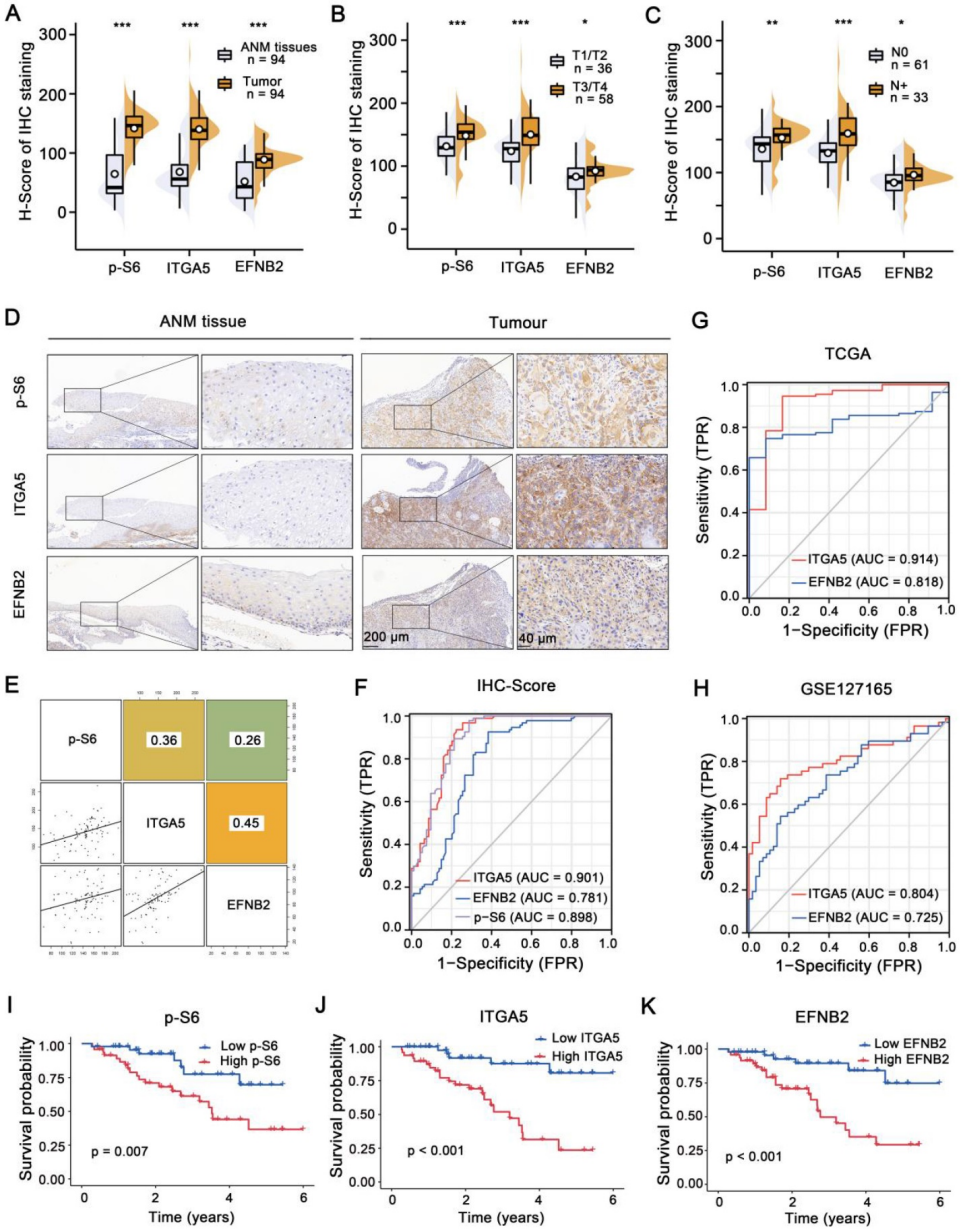
** Activation of the mTORC1-ITGA5-EFNB2 signaling pathway is associated with malignant progression and poor prognosis of LSCC. (A-C)** H-score statistical analysis of p-S6, EFNB2, and ITGA5 IHC staining from LSCC tissues and ANM tissues** (A)**, T1/T2 and T3/T4 LSCC tissues **(B)**, or with lymph node metastasis (N+) and non-lymph node metastasis (N0) LSCC tissues **(C)**. Error bars, mean ± SD. *P < 0.05, **P < 0.01; ***P < 0.001.** (D)** Representative IHC images of p-S6, ITGA5, and EFNB2 staining from the ANM tissues and LSCC tissues. Scale bar, 200 µm (low-magnification), 40 µm (high-magnification). **(E)** The correlation between p-S6, ITGA5, and EFNB2 expression of LSCC tissues. The square in the upper right corner demonstrates the Pearson's correlation value between the indicated genes. The scatterplot matrix fitted trend lines for the indicated genes are shown at the square in the lower left corner.** (F)** Diagnostic ROC curves of p-S6, ITGA5, and EFNB2 expression based on the H-scores of 94 LSCC tissues. **(G-H)** Diagnostic ROC curves of ITGA5, and EFNB2 expression based on the TCGA **(G)** and the GSE127165 datasets **(H)**. **(I-K)** Kaplan-Meier analysis of the association between overall survival and p-S6 **(I)**, ITGA5 **(J)**, or EFNB2 **(K)** protein levels in 94 LSCC patients. According to the median H-score of IHC staining, p-S6, ITGA5, and EFNB2 expression levels were divided into high (n = 47) and low (n = 47) subgroups.

IHC staining was employed to analyze p-S6, ITGA5 and EFNB2 expression levels in a retrospective cohort of 94 clinicopathologically characterized LSCC cases, including 14 cases of tumor stage 1 (T1, 14.9%), 22 cases of T2 (23.4%), 27 cases of T3 (28.7%), and 31 cases of T4 (33.0%) (Table S1-4). The protein expression levels were quantified by H-scores, and the results showed that p-S6, ITGA5, and EFNB2 protein levels in the LSCC tissues were significantly higher than those in the ANM tissues (Figure 7A and D). Analysis of the association between p-S6, ITGA5 or EFNB2 protein levels and the clinicopathological parameters in the 94 LSCC samples revealed that high expression levels of these three proteins positively correlated with T stages and cervical lymph metastasis (N stages) (Figure 7B-C).

Next, Pearson's correlation analysis was carried out, and the results showed that ITGA5, p-S6 and EFNB2 expression levels were positively associated with each other (Figure 7E). Furthermore, ROC curves were constructed based on the expression levels of ITGA5, EFNB2 and p-S6 in 94 LSCC tissues (AUC: 0.901, 0.781 and 0.898, respectively) (Figure 7F). ROC curve analysis was also performed using TCGA (AUC of ITGA5 and EFNB2: 0.914, 0.818) and GSE127165 (AUC of ITGA5 and EFNB2: 0.804, 0.725) databases (Figure 7G-H). All these results consistently suggest that ITGA5, EFNB2 or p-S6 in tumor tissues have good sensitivity and specificity as diagnostic markers for LSCC. In addition, according to median p-S6, ITGA5 or EFNB2 protein level, 94 LSCC samples were divided into high- (n = 47) and low-expression (n = 47) groups. As shown in Figure 7I-K, high p-S6, ITGA5, or EFNB2 expression predicted poor prognosis of LSCC patients. Collectively, the activated mTORC1-ITGA5-EFNB2 signaling pathway may play important roles in LSCC progression and prognosis.

### Depletion of ITGA5 potentiates the efficacy of CDDP

The combination of chemotherapy and RNA interference is a promising approach for efficient cancer therapy. To probe deeper into the clinical role of ITGA5 in LSCC, we first treated ITGA5 KO LIU-LSC-1 cells and the control cells with increasing concentrations of the chemotherapeutic drug CDDP *in vitro*. The results showed that knockout of ITGA5 significantly increased the sensitivity of LIU-LSC-1 cells to CDDP (Figure S9A). In contrast, overexpression of ITGA5 reduced the sensitivity of TU177 cells to CDDP (Figure S9B). Furthermore, LIU-LSC-1 cells were subcutaneously injected into the right flank of each BALB/c nude mice to establish a cell line-derived xenograft (CDX) tumor model. The tumor-bearing mice were treated with intratumoral injection of either ITGA5 siRNA or siNC, together with intraperitoneal injection of CDDP or normal saline (NS) for three weeks. As shown in Figure S9C-E, administration of ITGA5 siRNA attenuated the tumor growth of LIU-LSC-1 cells in the nude mice. Moreover, knockdown of ITGA5 by specific siRNAs significantly increased the antitumor activity of CDDP. IHC staining revealed that the proliferation marker Ki-67 and the angiogenic marker CD31 were decreased in treatment groups compared with the control group, which was consistent with the tumor burdens in different groups (Figure S9G). There was no body weight loss during the experimental period (Figure S9F).

**Figure 8:**
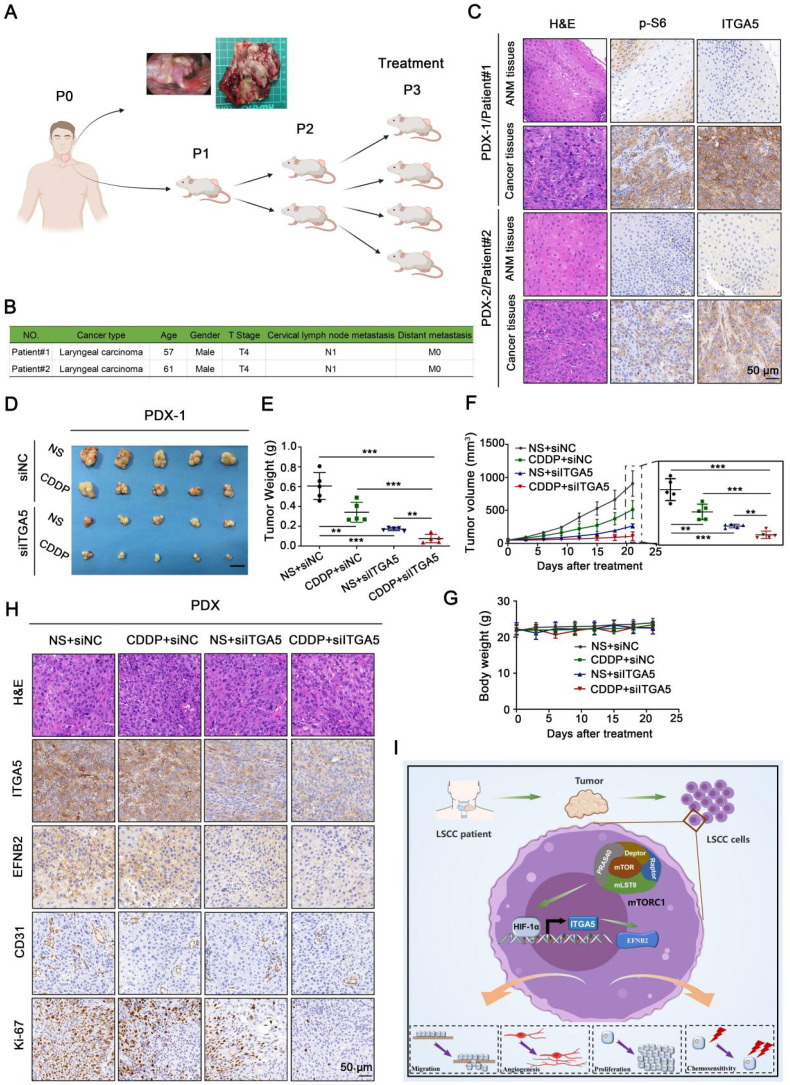
** Knockdown of ITGA5 increases chemosensitivity to CDDP in LSCC PDX models. (A)** Graphic illustration of the generation of LSCC PDX mouse models (Image partly made by BioRender.com.) **(B)** Clinical characteristics of donor patients. **(C)** Representative H&E images and IHC images of ITGA5 and p-S6 staining from ANM or tumor tissues of donor patients. Scale bar, 50 µm. **(D-F)** Tumor images **(D)**, tumor weight **(E)**, and tumor volume **(F)** of PDX model tumors with the indicated treatment. Scale bars, 1 cm **(D)**. **(G)** Body weight of the mice. Error bars, mean ± SD (n = 5 mice/group). **P < 0.01; ***P < 0.001. **(H)** Representative IHC images of ITGA5, EFNB2, CD31, and Ki-67 staining from the indicated PDX model tumors. Scale bar, 50 µm. **(I)** Schematic illustration of the activated ITGA5-EFNB2 axis is critical for mTORC1-mediated tumor proliferation, angiogenesis, and metastasis of LSCC.

The above experimental results prompted us to further study the combination effect of ITGA5 siRNAs and CDDP against LSCC in PDX models. Precisely, fresh tumor tissues were isolated from two patients with LSCC, and of these tumors the one with more activated mTORC1 and higher ITGA5 expression was chosen to establish the PDX model (Figure 8A-C). Similar to the CDX models, the growth of tumor size and weight in ITGA5 siRNAs group was remarkably retarded compared with that in the siNC group. Moreover, tumors in ITGA5 siRNAs group were significantly more sensitive to CDDP treatment compared with those in the siNC group (Figure 8D-F), and no significant body weight changes were found during the experimental period (Figure 8G). Successful *in vivo* knockdown of ITGA5 in tumors by siRNAs, along with the downregulation of its target EFNB2, was confirmed by IHC analysis. Furthermore, IHC staining of Ki-67 and CD31 in the tumors of the PDX models showed a similar pattern to that of the CDX models (Figure 8H). In summary, silencing of ITGA5 increases chemosensitivity to CDDP in mTORC1-activated LSCC cells.

## Discussion

Immortalized cancer cell lines are valuable models for elucidating the molecular mechanism of carcinogenesis and developing effective therapeutic strategies against cancer. Nevertheless, many cell lines often lose their original biological characteristics and are likely to become contaminated with microorganisms during long-term culture *in vitro* 27, 28. Thus, the establishment of novel cell lines that can effectively reflect clinical features of cancer patients is urgently needed. Specifically, very few established LSCC cell lines can be obtained through public channels as compared to other types of epithelial-derived tumor cell lines. In this study, a new LSCC cell line (LIU-LSC-1) was established from the primary tumor derived from an untreated 74-year-old male patient with LSCC and cervical lymph node metastases. As of this writing, this cell line has been maintained for more than 100 passages *in vitro* without showing changes in morphology or proliferative potential. The high activation of mTORC1 represents a crucial characteristic of LIU-LSC-1 cells, which may give priority to the growth and proliferation of cells. LIU-LSC-1 cells exhibited pronounced tumor formation and metastatic abilities. Thus, this mTORC1-hyperactived LSCC cell line may provide researchers with a novel model to investigate the molecular pathogenesis of LSCC.

Accumulating studies indicate that the expression of ITGA5 is abnormally elevated in many types of human cancers, which may contribute to cancer cell proliferation and metastasis 29-32. However, the specific function and upstream regulatory signaling of ITGA5 in LSCC are relatively obscure. In this study, mainly based on our newly established LSCC cell line (LIU-LSC-1) and TU177 cells, LSCC tissue samples, and high-throughput RNA-sequencing data from TCGA and Gene Expression Omnibus (GEO), we proposed that ITGA5 is a new downstream target of mTORC1 signaling in LSCC cells. Depletion of ITGA5 attenuated cell proliferation, migration, angiogenesis, tumor growth, and tumor metastasis, whereas overexpression of ITGA5 exerted the opposite effect. The clinical data analysis further indicated that ITGA5 positively correlated with mTORC1 activity in LSCC, and that increased expression of ITGA5 was associated with advanced clinicopathologic characteristics (clinical stage, metastasis) and poor prognosis. More importantly, knockdown of ITGA5 suppressed LSCC tumor growth and sensitized LSCC to CDDP therapy in our CDX and PDX models. In addition, the transcription factor HIF-1α, a well-known target of mTORC1, was identified to mediate the transcription of ITGA5 in LSCC cells, which is in agreement with previous findings that ITGA5 is a hypoxia inducible gene in many cell lines 33-36. Our findings not only suggest that ITGA5 exerts a vital role in the tumorigenesis of LSCC, which is consistent with the result of a more recent bioinformatics analysis of LSCC performed by Zhou et al. 37, but also indicate mTORC1 signaling as an important upstream regulator of ITGA5 (Figure 8I). Therefore, ITGA5 is a potential anticancer target for hyperactivated mTORC1-related LSCC. What's more, depletion of ITGA5 markedly attenuated the activity of AKT and mTORC1 while overexpression of ITGA5 enhanced their activity (Figure S10A-B). It thus may be anticipated that there was a positive feedback loop between mTORC1 and ITGA5 and ITGA5 specific inhibitors can overcome the defect of rapamycin in anti-tumor therapy of mTORC1-related LSCC. In addition to LSCC cells, hyperactivated mTORC1 also led to increased expression of ITGA5 in Tsc1- or Tsc2-null MEFs (Figure S11A-B) -the most widely used cell models for the study of mTORC1 signaling 38, 39. Thus, it appears to be ubiquitous across species that mTORC1 upregulates ITGA5. The mTORC1-ITGA5 signaling pathway may play a key role in other cancers besides LSCC.

ITGA5 primarily binds to integrin subunit beta 1 (ITGB1) to form a heterodimer; the heterodimer can recognize and adhere to extracellular ligands containing RGD tripeptide motif, and then subsequently modulate multiple cellular biological activities through activating several signaling pathways, such as FAK, AKT, and MAPK signaling 40. However, the specific downstream molecules of ITGA5 remain largely unknown. In this study, we identified EFNB2 as a novel functional downstream target of ITGA5 in LSCC cells based on multiple lines of evidence. First, knockout of ITGA5 reduced EFNB2 expression, while ectopic expression of ITGA5 enhanced EFNB2 expression. Second, EFNB2 possessed ITGA5-mimicking behavior. Third, overexpression of EFNB2 rescued, at least in part, ITGA5 knockout-mediated reduction of tumor cell proliferation, angiogenesis, and metastasis in LSCC cells, whereas knockdown of EFNB2 attenuated the above cell behaviors driven by ITGA5 overexpression. Finally, in line with ITGA5, EFNB2 was elevated in LSCC tumor tissues, predicted lymph node metastasis, and individuals with poor overall survival, and its expression levels were associated with mTORC1 activities and ITGA5 expression levels in primary tumors.

As a cell surface transmembrane ligand for Eph receptors, EFNB2 plays an important role in migration, invasion, and angiogenesis of cancer cells 41-43. Consistent with our findings, it has been shown that EFNB2 is expressed at abnormally high levels in HNSCC and that its level of expression is related to the malignant progression of the tumor 44. Blockade of EFNB2 in PDX tumor derived from a patient with recurrent oral cavity cancer inhibited tumor proliferation and increased animal survival 44. Our study not only highlights the tumor supportive role of EFNB2 in LSCC but also demonstrates that EFNB2 is a substantial effector of the mTORC1-ITGA5 pathway (Figure 8I). However, it remains to be addressed how ITGA5 regulates the transcription of EFNB2 mRNA. The expression of EFNB2 is upregulated by Notch signaling 45, 46. Our previous study has shown that mTORC1 positively regulates Notch signaling in mouse and human cells through upregulation of Jagged1 47. As shown in Figure S12A, Jagged1 was correlated with ITGA5 and EFNB2 by analysis of the TCGA and GSE 127165 datasets. Both the knockout and knockdown of ITGA5 inhibited the expression of Jagged1 and downregulated the Notch signaling (detected by NICD and Hes1 levels) in the LIU-LSC-1 and TSC2 KO1 TU177 cells, while overexpression of ITGA5 upregulated the Jagged1-Notch signaling in the TU177 cells (Figure S12B-C). Furthermore, inhibition of the Notch signaling with DAPT suppressed EFNB2 expression in the LIU-LSC-1 and TSC2 KO1 TU177 cells (Figure S12D). In contrast, activation of the Notch signaling by Jagged1-Fc resulted in an opposite effect on the EFNB2 levels in the TU177 cells (Figure S12E). Therefore, it is likely that elevated ITGA5, resulting from hyperactivated mTORC1, enhances EFNB2 expression by the activation of Jagged1-Notch signaling pathway in LSCC cells.

In summary, our study proved that ITGA5 is a novel target of mTORC1 in LSCC cells. ITGA5 is upregulated in LSCC tissues, indicating a gloomy prognosis, and it is positively correlated with mTORC1 signaling in LSCC patients. Upregulated ITGA5 increases EFNB2 expression, leading to the promotion of LSCC progression. Our studies emphasized the importance of the mTORC1-ITGA5-EFNB2 pathway in the progression of LSCC, suggesting that the ITGA5-EFNB2 pathway may serve as a prognostic biomarker and potential therapeutic target for mTORC1-related LSCC.

## Supplementary Material

Supplementary materials and methods, figures, and tables 1-8.

Supplementary table 9.

Supplementary table 10.

Supplementary table 11.

## Abbreviations

ANM: adjacent normal mucosal
AJCC: the American Joint Committee on Cancer
CAFs: Cancer-associated fibroblasts
CAM: Chicken chorioallantoic membrane assay
CCK-8: Cell Counting Kit-8
CDDP: cisplatin
CDX: cell line-derived xenograft model
ChIP: Chromatin immunoprecipitation
co-DEGs: common differential genes
DEGs: differentially expressed genes
DFX: deferoxamine
EDD: embryonic developmental day
FBS: fetal bovine serum
EFNB2: ephrin-B2
GEO: Gene Expression Omnibus
H&E: hematoxylin and eosin
HIF-1α: hypoxia-inducible factor 1α
HPV: human papilloma virus
IF: immunofluorescence
IHC: Immunohistochemical
ITGA5: integrin subunit alpha 5
ITGB1: integrin subunit beta 1
LSCC: Laryngeal squamous cell carcinoma
MEFs: murine embryonic fibroblasts
mTOR: Mechanistic target of rapamycin
mTORC1: Mechanistic target of rapamycin complex 1
PBS: phosphate-buffered saline
PDX: patient-derived xenograft model
qRT-PCR: quantitative real-time PCR
RAD001: everolimus
Rapa: rapamycin
ROC: receiver operating characteristic
sgRNAs: single-guide RNAs
shRNAs: short hairpin RNAs
siRNAs: small interfering RNAs
STR: short-tandem repeat
TCGA: The Cancer Genome Atlas
