# Supplementary material for: Elevated ITGA5 facilitates hyperactivated mTORC1-mediated progression of laryngeal squamous cell carcinoma via upregulation of EFNB2
Source: Theranostics. 2022 Oct 24;12(17):7431–49. doi: 10.7150/thno.76232 (PMC9691358; doi:10.7150/thno.76232)
Supplement: Supplementary file 1 — Supplementary materials and methods, figures, and tables 1-8. [file thnov12p7431s1.pdf]

Elevated ITGA5 facilitates hyperactivated mTORC1-mediated progression of laryngeal squamous cell carcinoma via upregulation of EFNB2

MATERIALS AND METHODS

Tumor specimens

A total of 94 LSCC and adjacent normal mucosal (ANM) tissues were acquired during routine surgeries at the First Affiliated Hospital of Anhui Medical University (Anhui, China) from 2014 to 2020. None of the patients were subjected to chemotherapy, radiotherapy, or other related antitumor therapies before surgery. The TNM staging was done referring to the American Joint Committee on Cancer (AJCC) 8th edition TNM Staging Criteria. The study was conducted in accordance with Declaration of Helsinki, and the ethical approval was obtained from the First Affiliated Hospital of Anhui Medical University Research Ethics Committee. All of the patients provided a written informed consent before participation. Detailed information of all the 94 LSCC patients is listed in Table S1-4.

Establishment and characterization of a novel LSCC cell line, LIU-LSC-1

Fresh tumor tissue was isolated from a 74-year-old LSCC patient (T3N1M0) who underwent surgery, and the specimen was immediately immersed in RPMI 1640 medium (Gibco，NY，USA） containing penicillin (100 U/mL)/streptomycin (0.1 mg/mL) and amphotericin B (0.25 μg/mL) (Beyotime, Jiangsu, China). The tissue sample was washed three times in phosphate-buffered saline (PBS) and cut into small pieces. Then, small tumor masses were dissociated enzymatically in RPMI 1640

medium containing 200 U/mL type IV collagenase (Sigma, Saint Louis, MO, USA) at 37 °C for 12 h. After two rounds of washing in PBS and centrifugation, the sediments were seeded onto 60 mm Petri dishes and cultured in Epithelial Cell Complete Medium (VivaCell, Shanghai, China) with 1% penicillin/streptomycin (Beyotime). After 3 days of incubation, the cell culture medium was replaced. Cells were passaged every 3 to 4 days. Cancer-associated fibroblasts (CAFs) were removed by a brief exposure to trypsin digestion (0.25% trypsin-EDTA, Beyotime). Cells were named LIU-LSC-1 and compared with the short-tandem repeat (STR) data of cell lines included in ATCC, DSMZ, JCRB and RIKEN databases. No closely matched cell lines were found (Table S5, EV < 0.8), which suggested that LIU-LSC-1 may be a new cell line. Mycoplasma analysis of this cell line was negative.

For Giemsa staining, followed by fixation with methanol for 10 min, LIU-LSC-1 cells were stained with crystal violet for 10 min and photographed under microscope. Ultrastructural analysis of cells was performed by transmission electron microscopy (TEM). In brief, LIU-LSC-1 cells were fixed with 2.5% glutaraldehyde at 4 °C for 2.5 h, and then the cells were washed three times with PBS and post-fixed in 1% OsO_4 for 2 h at 4 °C. After being dehydrated through an ethanol gradient, the samples were embedded in Spurr's resin. Then ultrathin sections were cut and stained with either uranyl acetate or lead citrate. The samples were observed under a JEOL JEM1400 TEM. Tonofilaments in the cytoplasm, desmosomes in the intercellular connections, intranuclear inclusions and indented nuclear membrane are supposed to the characteristics of tumor cells [1].

For flow cytometry, LIU-LSC-1 cells at the logarithmic growth phase were stained with CD44 (5 μg/mL) or the isotype control antibodies (5 μg/mL) for 30 min at room temperature in the dark. After washed three times by centrifugation-resuspension with ice-cold PBS, the cells were stained with a secondary antibody (Goat Anti-Mouse IgG H&L, DyLight® 488) and incubated for 30 min. Subsequently, the cells were analyzed by flow cytometer (Becton Dickinson, San Diego, CA, USA).

Cell culture

Cell source and culture conditions of murine embryonic fibroblasts (MEFs) ($Tsc1^{+/+}$, $Tsc1^{-/-}$, $Tsc2^{+/+}$, and $Tsc2^{-/-}$), HEK293T cells and LSCC cell lines (AMC-HN-8, TU177, and LIU-LSC-1) are listed in Table S6. MEFs ($Tsc1^{+/+}$, $Tsc1^{-/-}$, $Tsc2^{+/+}$, and $Tsc2^{-/-}$) have been described previously [2, 3]. For hypoxic exposure, cells were cultured under hypoxic (1% O_2) or normoxic (21% O_2) conditions for the indicated times. All cell lines were verified by STR analysis and tested for mycoplasma contamination by MycoAlert Mycoplasma Detection Kit (Lonza #LT07-118).

Antibodies, reagents and plasmids

All information regarding antibodies used in this study is provided in Table 7. Rapamycin (Rapa), everolimus (RAD001), deferoxamine (DFX), DAPT and MHY1485 were purchased from Selleck Chemicals (Houston, TX, USA). Jagged1-Fc was obtained from R&D system (Minneapolis, MN, USA). Lipofectamine RNAiMax

was obtained from Invitrogen (Carlsbad, CA, USA). pRL-TK, pGL3-Basic, pcDNA3.0, pcDNA3.0-HA-HIF-1α, lenti-CRISPRv2 plasmids and packaging vectors (pVSVG and psPAX2) were purchased from Addgene (Cambridge, MA, USA).

RNA interference, lentivirus infection and CRISPR-Cas9

LIU-LSC-1 cells were seeded into 12-well plates and transfected with small interfering RNAs (siRNAs) using Lipofectamine RNAiMax (GenePharma, Shanghai, China). The sequences used are as follows: mTOR 5'-CCCUGCCUUUGUCAUGCCUTT-3'; Rictor, 5'-ACUUGUGAAGAAUCGUAUCTT-3'; Raptor, 5'-GGACAACGGCCACAAGUACTT-3'; Negative control (NC), 5'-UUCUCCGAACGUGUCACGUTT-3'.

LIU-LSC-1 cells were stably infected with short hairpin RNAs (shRNAs) targeting HIF-1α, Raptor, ITGA5 and EFNB2 using lentivirus vector GV248 (GenePharma). The target sequences used are as follows: shRaptor-1, 5'-GGACAACGGCCACAAGTAC-3'; shRaptor-2, 5'-CCCTCATCGGAGTTTCCTT-3'; shHIF-1α-1, 5'-GCCGCTCAATTTATGAATA-3'; shHIF-1α-2, 5'-GCTGGAGACACAATCATAT-3'; shITGA5, 5'-GCTACCTCTCCACAGATAACT-3'; shEFNB2-1, 5'-GCAGAACTGCGATTTCCAAAT-3'; shEFNB2-2, 5'-GGAATTCCTCGAACTCCAAAT-3'; the control scrambled shRNA (shSc), 5'-TTCTCCGAACGTGTCACGT-3'. The recombinant vectors were co-transfected with packing vectors (psPAX2 and pVSVG) into HEK293T cells. After 48 h, the viral

supernatants were filtered and used to infect LIU-LSC-1 cells. ITGA5 and EFNB2 overexpressing cell lines were generated by lentivirus vector GV492 containing the full-length cDNA sequence of human ITGA5 and EFNB2, respectively (GenePharma).

The following single-guide RNAs (sgRNAs) targeting ITGA5 and TSC2 were designed by Open-access software program CRISPR and synthesized by Sangon Biotech Co., Ltd. (Shanghai, China): ITGA5-sgRNA#1, 5'-GGGGCAACAGTTCGAGCCCA-3'; ITGA5-sgRNA#2, 5'-GGAGCCACTGAGCGACCCCG-3'; TSC2-sgRNA, 5'-CACCGAACAATCGCATCCGGATGAT-3'. Oligos were then cloned into the Cas9 backbone Lenti-CRISPRv2 vector. Recombinant plasmids, psPAX2 and pVSVG were co-transfected into HEK293T cells. The medium was harvested and filtered to remove cell debris 48 h later. After infection, the cells were obtained by culture over 14 days in 1.5 μg/mL puromycin (Sigma, MO, USA). Then cells were placed in 96-well plates and examined by microscopy the next day to be sure that only one cell was seeded per well. Clones were passaged after 10 days and monoclonal lines were screened via western blotting for ITGA5 knockout.

Total RNA isolation, quantitative real-time PCR (qRT-PCR) assay and RNA sequencing

Total RNA extraction, cDNA synthesis, qRT-PCR and RNA sequencing were performed as described previously [4]. The primers for qRT-PCR (provided by

Sangon Biotech Co., Ltd.) are shown in Table S8. The RNA samples were sequenced on Illumina Novaseq™ 6000 (LC Sciences, Hangzhou, China).

Western blot analysis

The total protein of cells was harvested using RIPA lysate (Beyotime). The lysates were separated by NuPAGE 4-12% Bis-Tris and transferred to polyvinylidene fluoride membrane (Millipore, Billerica, MA, USA). Then the membrane was blocked with 5% nonfat milk for 1 h and incubated with the primary antibodies (diluted 1: 1000) at 4 °C overnight. Final detection was performed using chemiluminescence after the secondary antibody incubation.

Chromatin immunoprecipitation (ChIP)

The ChIP assay was performed as previously described using a SimpleChIP® Plus Enzymatic Chromatin IP Kit (Cell Signaling Technology, MA, USA) [5]. PCR primer sequences for the putative HIF-1α-binding region (PBR) and a nonspecific HIF-1α-binding region (NBR) of human ITGA5 were as follows: Site1, forward, 5'-CCACCCCTAATCTCCCAAATCCT-3'; reverse, 5'-TCAGGATCTTTAAGCCCAGCATTG-3'; Site2, forward, 5'-CCAAACCCGCCCAGTCTAACC-3'; reverse, 5'-GGGGGGCATTCCTGGGT-3'; NBR, forward, 5'-CAAAGCCAGCACCAGTGAAGAGAC-3'; reverse, 5'-CCCTCCTCCCAACACACACATATATAC-3'. The primer sequences for qRT-PCR were as follows: NBR, forward, 5'-AAGCCAGCACCAGTGAAGAGAC-3'; reverse, 5'-ACTCCTGGTTCTAGCTACTTTAATCAC-3'. PBR, forward,

5'-CGCCCAGTCTAACCCAGTCCA-3'; reverse, 5'-CCTGGGTCCCTGGAACTCTGAG-3'.

Reporter constructs and luciferase reporter assay

A 334-bp fragment of the human ITGA5 promoter (-266/+67) containing the intact HIF-1α-binding site was obtained by PCR using human genomic DNA. The primer sequences were as follows: forward, 5'-GGGGTACCTGGAAAGGAATGGGGAGGAAGGAG-3'; reverse, 5'-GAAGATCTGCGCCCGCTCTTCCCTGTCC-3'. The fragment was cloned into the *Bgl* II and *Kpn* I sites of the pGL3-Basic plasmid (ITGA5-Luc). A Q5® Site-Directed Mutagenesis Kit (NEB, Ipswich, MA, USA) was used to mutate the potential HIF-1α-binding site (ITGA5-mut). The primer sequences were as follows: forward, 5'-CCCCTAAGGGAAATGGGGGGGGGCGC-3'; reverse, 5'-TGGGGGACGCGGGCTCAG -3'. The 293T cells were then seeded into 24-well culture plates and transfected with 400 ng of ITGA5-Luc or ITGA5-mut together with 20 ng of an internal control plasmid pRL-TK and 400 ng of HA-HIF-1α-pcDNA3.0 or the empty vector pcDNA3.0. The luciferase activity was estimated using the Dual-Luciferase Reporter Assay System (Promega, USA).

***In vitro* functional assays**

Cell Counting Kit-8 (CCK-8, TargetMol, Shanghai, China), colony formation, wound healing and transwell assays were used to test the in vitro functional roles of ITGA5 and EFNB2. For CCK-8 assay, cells were seeded onto 96-well plates with

indicated treatment at 1000 cells/well. 10 μL of CCK-8 reagent was added to each well at the specified time point. After incubation for 2 h, the reaction product was measured at 450 nm using a microplate reader. For colony formation assays, 1500 of treated cells were seeded into 60 mm plates. 10 days later, cells were fixed with 4% paraformaldehyde and stained with crystal violet. Colonies containing more than 50 cells were counted. For wound healing assay, in order to create a narrow wound-like gap, the monolayer of cells was scratched with a 200-μL pipette tip. Cell migration into the wound area was recorded for each condition after 18 h or 24 h. For transwell assays, 24-well transwell chambers (Corning, NY, USA) were used. 2×10^4 cells in 200 μL of DMEM (or RPMI 1640) with 1% FBS were seeded in the upper chamber and the lower chamber contained 500 μL medium with 10% FBS. The chambers were incubated at 37 °C with 5% CO_2 for 24 h (migration assay, without matrigel; invasion assay, coated with 250 μg/mL matrigel coating). Then, cells on the upper surface of the filter were removed and the cells on the lower membrane surface were stained with crystal violet after fixation with 4% paraformaldehyde. Cell migration and invasion were quantified by counting 10 random fields under a microscope (200×).

Chicken chorioallantoic membrane (CAM) assay

Pathogen-free fertilized chicken eggs were purchased from Jinan SAIS Poultry Company (Shangdong, China). The CAM assay was performed as described previously [6]. Briefly, On embryonic developmental day 8 (EDD 8), a window about 1.0 cm was opened in the shell of each egg, and sterile gelatin sponge mixed with 20 μL of cell suspension containing 2×10^6 LSCC cells was planted on CAM. The CAM

was separated from the eggs after fixation with stationary solution (methanol: acetone, 1:1) for 30 min on EDD 15. Then, the CAM was recorded by a digital camera, and the number of blood vessels that converged toward the implant were counted by three blind observers.

Animal experiments, cell derived xenograft (CDX) models and patient-derived xenograft (PDX) models

All animal studies were performed under approval of the Experimental Animal Ethical Committee of Anhui Medical University. Male BALB/c nude mice and NOD/SCID mice (4-week-old) were purchased from GemPharmatech Co., Ltd (Nanjing, China). For tumorigenicity assays, 5×10^6 genetically engineered LSCC cells were subcutaneously injected into the right armpits of each mice (five per group), respectively. The tumor volume was measured and calculated by the formula $V = 0.5 \times W^2 \times L$ (V, volume; L, length; W, width).

For tumor metastasis experiments, 1×10^6 genetically engineered LSCC cells suspended in 100 μL PBS were injected into nude mice via the tail vein. Mice were killed and metastatic lung tumors were analyzed under dissecting microscope after hematoxylin and eosin (H&E) staining at 8 weeks after tail vein injection.

For intratumoral siRNAs injections, the chemically modified siRNAs were provided by GenePharma. The target sequences are listed as follows: siNC, 5'-UUCUCCGAACGUGUCACGUTT-3'; siITGA5-1, 5'-UACCUCUCCACAGAUAACUTT-3'. EntransterTM-*in vivo* transfection reagents were provided by Engreen Biosystem Co., Ltd (Beijing, China).

For CDX models, 200 μL serum-free RPMI 1640 containing 5 × 10^6 LIU-LSC-1 cells were subcutaneously injected into the right flank of each mouse. After tumors were detectable, 20 tumor-bearing mice were randomly assigned into four groups (five per group) and were treated with CDDP (3 mg/kg, twice/week), normal saline (NS, twice/week), together with ITGA5 siRNAs (100 μg, twice/week) or non-targeting control siRNAs (100 μg, twice/week). siRNA was injected directly into the tumor bodies at two or more spots each time. NS and CDDP were injected into the abdominal cavity. The mice were sacrificed after 3 weeks of treatment, and then tumors were dissected and weighed. Furthermore, tissues were embedded in paraffin for H&E or IHC.

For PDX models, freshly excised tumor tissues were obtained from a LSCC patient receiving surgery at the First Affiliated Hospital of Anhui Medical University. The tissues were cut into 2 × 2 × 3-mm^3 pieces (kept in PRMI 1640 supplemented with penicillin and streptomycin) and grafted subcutaneously into the flank of NOD/SCID mice as P1. PDX tumors were harvested and transplanted into BALB/c nude mice as P2 when the tumor size upon reached a size of 1000 mm^3. We followed the aforementioned protocols to transplant PDX tumor tissues into next-generation mice as P3 and performed next step according to the protocol of CDX models.

Immunohistochemical staining (IHC) and immunofluorescence (IF) assay

IHC analysis staining was performed as previously described [5]. Antibodies against ITGA5 (diluted 1:50), EFNB2 (diluted 1:200), p-S6 (diluted 1:75), Ki-67 (diluted 1:100) and CD31 (diluted 1:100) were used. A modified histologic score

(H-scores, [{% of weak staining} × 1] + [{% of moderate staining} × 2] + [{% of strong staining} × 3]) was used to evaluate IHC staining [7, 8]. Each staining obtained an H-score between 0 and 300, and the average of H-score for all the cases was calculated.

For IF assays, Cells were treated with DMSO, Rapa (20 nM), RAD001 (50 nM) or MHY1485 (10 μM) for 24 h and then stained as previously described [5]. Primary antibodies against ITGA5 (diluted 1:50), EFNB2 (diluted 1:200), or CD44 (diluted 1:1000) and FITC-conjugated secondary antibody (diluted 1:1000) were used. DAPI (Beyotime) was used to stain nuclei. The images were captured by LSM880 + Airyscan confocal laser scanning microscope (Carl Zeiss, Oberkochen, Germany).

Bioinformatics analysis and Statistical analysis

RNA sequencing data and clinical information of LSCC were obtained from the Gene Expression Omnibus (GEO) dataset (http://www.ncbi.nlm.nih.gov/geo/) and The Cancer Genome Atlas (TCGA) (http://cancergenome.nih.gov/). The receiver operating characteristic (ROC) curves were used to evaluate the sensitivity and specificity of genes as diagnostic biomarkers.

All statistical analyses were performed using GraphPad Prism 6.0. Differences between two experimental groups were conducted using the two-tailed Student's t-test. Correlations between genes were analyzed by Pearson's correlation analysis. The survival rates were calculated by the Kaplan-Meier method. $P < 0.05$ was considered statistically significant (*$P < 0.05$, **$P < 0.01$ and ***$P < 0.001$).

References

1. Wu CP, Zhou L, Gong HL, Du HD, Tian J, Sun S, et al. Establishment and characterization of a novel HPV-negative laryngeal squamous cell carcinoma cell line, FD-LSC-1, with missense and nonsense mutations of TP53 in the DNA-binding domain. Cancer letters. 2014; 342: 92-103.

2. Zhang H, Bajraszewski N, Wu E, Wang H, Moseman AP, Dabora SL, et al. PDGFRs are critical for PI3K/Akt activation and negatively regulated by mTOR. The Journal of clinical investigation. 2007; 117: 730-8.

3. Zha X, Wang F, Wang Y, He S, Jing Y, Wu X, et al. Lactate dehydrogenase B is critical for hyperactive mTOR-mediated tumorigenesis. Cancer research. 2011; 71: 13-8.

4. Wang YN, Xu YF, Liang YX, Fan XY, Zha XJ. Transcriptomic Sequencing of Airway Epithelial Cell NCI-H292 Induced by Synthetic Cationic Polypeptides. BioMed research international. 2019; 2019: 3638469.

5. Wan X, Zhou M, Huang F, Zhao N, Chen X, Wu Y, et al. AKT1-CREB stimulation of PDGFRα expression is pivotal for PTEN deficient tumor development. Cell Death Dis. 2021; 12: 172.

6. Chen X, Miao M, Zhou M, Chen J, Li D, Zhang L, et al. Poly-L-arginine promotes asthma angiogenesis through induction of FGFBP1 in airway epithelial cells via activation of the mTORC1-STAT3 pathway. Cell Death Dis. 2021; 12: 761.

7. Detre S, Saclani Jotti G, Dowsett M. A "quickscore" method for immunohistochemical semiquantitation: validation for oestrogen receptor in breast carcinomas. J Clin Pathol. 1995; 48: 876-8.

8. Paschalis A, Sheehan B, Riisnaes R, Rodrigues DN, Gurel B, Bertan C, et al. Prostate-specific Membrane Antigen Heterogeneity and DNA Repair Defects in Prostate Cancer. Eur Urol. 2019; 76: 469-78.

SUPPLEMENTARY FIGURES

Figure S1

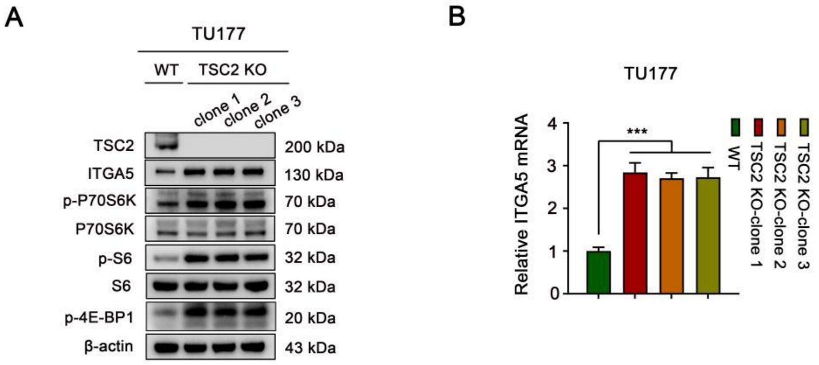

Figure S1. Knockout of TSC2 led to the activation of mTORC1 and the upregulation of ITGA5 in the TU177 cells. (A-B) TSC2 knockout TU177 cell lines were constructed using a CRISPR/Cas9 approach and three different clones were screened out. Cell lysates of the indicated cells were subjected to western blotting with the indicated antibodies **(A)**. ITAG5 mRNA levels in the indicated cells were detected using qRT-PCR **(B)**. The error bars represent the mean ± SD of triplicate technical replicates. ***P < 0.001.

Figure S2

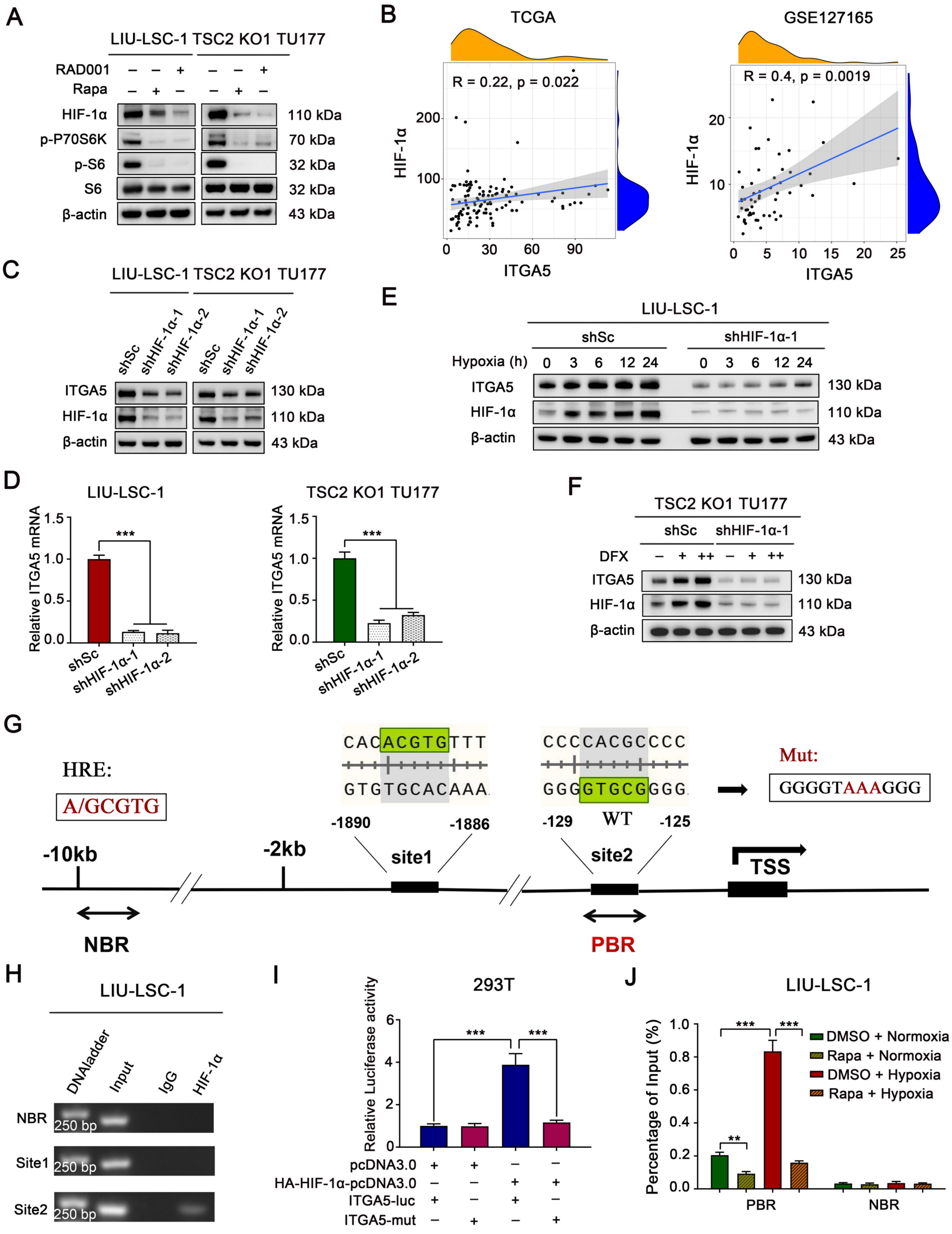

Figure S2. mTORC1 enhances ITGA5 expression through upregulation of HIF-1α. (A) LIU-LSC-1 and TSC2 KO1 TU177 cells were treated with 20 nM Rapa or 50 nM RAD001 for 24 h, and cell lysates were subjected to western blot analysis. **(B)** The correlation between HIF-1α and ITGA5 expression was analyzed by

Pearson's correlation analysis using the TCGA and GSE127165 cohorts. **(C-D)** LIU-LSC-1 and TSC2 KO1 TU177 cells were transfected with shRNAs targeting HIF-1α (shHIF-1α) or control shRNA- (shSc). Immunoblotting **(C)** and qRT-PCR **(D)** were performed to detect the expression of ITGA5. **(E-F)** LIU-LSC-1-shHIF-1α and LIU-LSC-1-shSc cells were treated with 1% O_2 for the indicated times **(E)**; HIF-1α-knockdown TSC2 KO1 TU177 cells and the control cells were treated with DFX (+ indicates 100 μM; ++ indicates 200 μM) for 24 h **(F)**. Cell lysates were subjected to western blot analysis **(E-F)**. (G) Schematic representation of the putative HIF-1-binding site of the human *ITGA5* gene. **(H)** LIU-LSC-1 cells were subjected to ChIP assay using an anti-HIF-1α antibody. Normal rabbit IgG antibody was used as a negative control. PCR amplifications were performed using primers surrounding the putative HIF-1α binding site of the human *ITGA5* gene. **(I)** The ITGA5-Luc or ITGA5-mut constructs together with HA-HIF-1α-pcDNA3.0 or pcDNA3.0 were co-transfected into the 293T cells with pRL-TK plasmid for 24 h, and then luciferase activity was estimated. **(J)** LIU-LSC-1 cells were treated with DMSO or 20 nM Rapa under normoxia or hypoxia condition for 24 h. HIF-1α antibody-immunoprecipitated DNA from these cells was amplified and quantified by qRT-PCR for NBR and PBR regions. The data were plotted as the ratio of immunoprecipitated DNA subtracting nonspecific binding to IgG vs. total input DNA (%). The error bars represent the mean ± SD of triplicate technical replicates. ***$P < 0.001$.

Figure S3

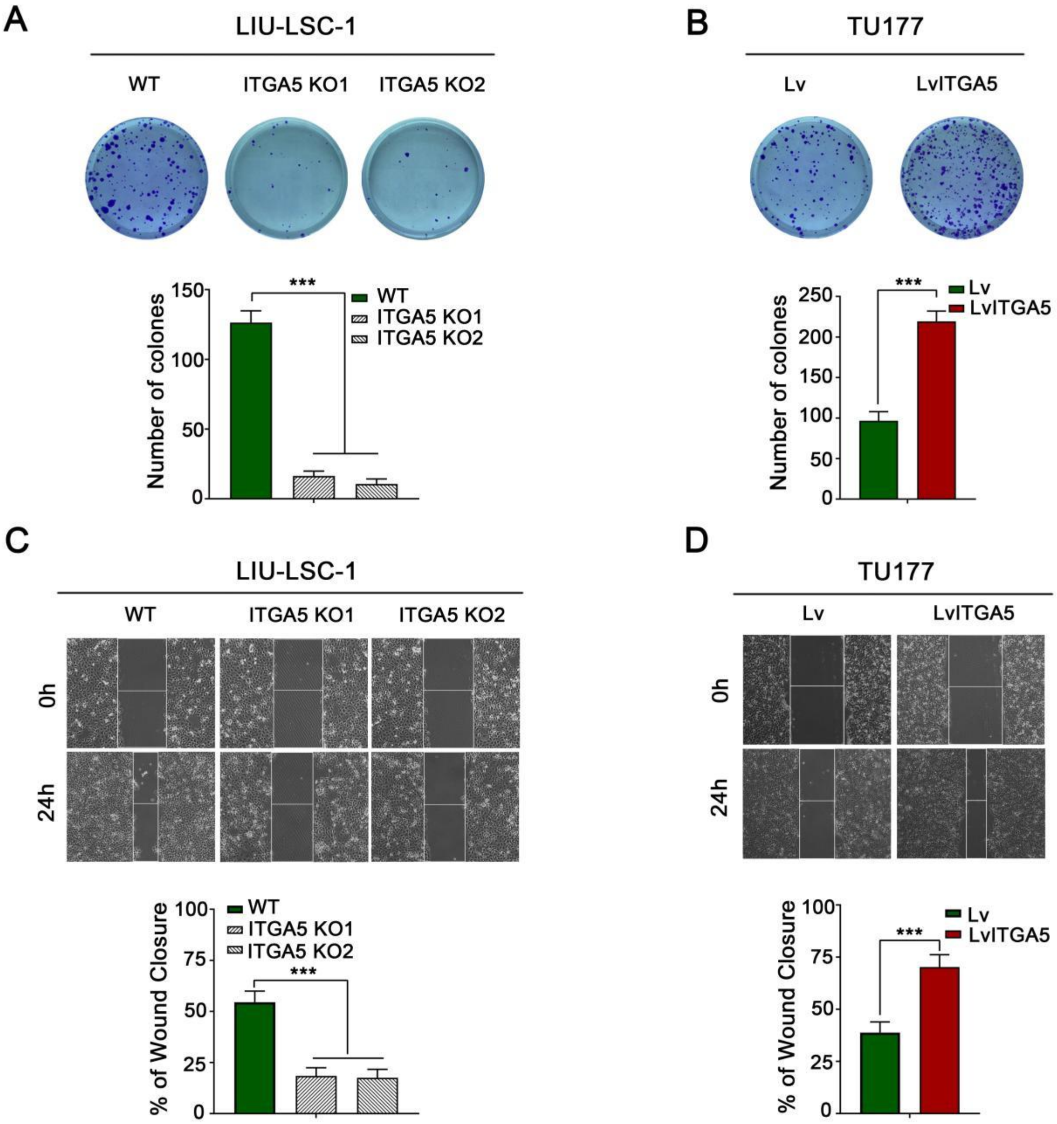

Figure S3. ITGA5 promotes proliferation and migration of LSCC cells. (A, C) ITGA5 WT and KO LIU-LSC-1 cells. **(B, D)** ITGA5-overexpressing TU177 cells and the control cells. Proliferative and migratory abilities of the indicated cells were measured by colony formation **(A-B)** and wound healing assays **(C-D)**, respectively. The error bars represent the mean ± SD of triplicate independent experiments. ***$P < 0.001$.

Figure S4

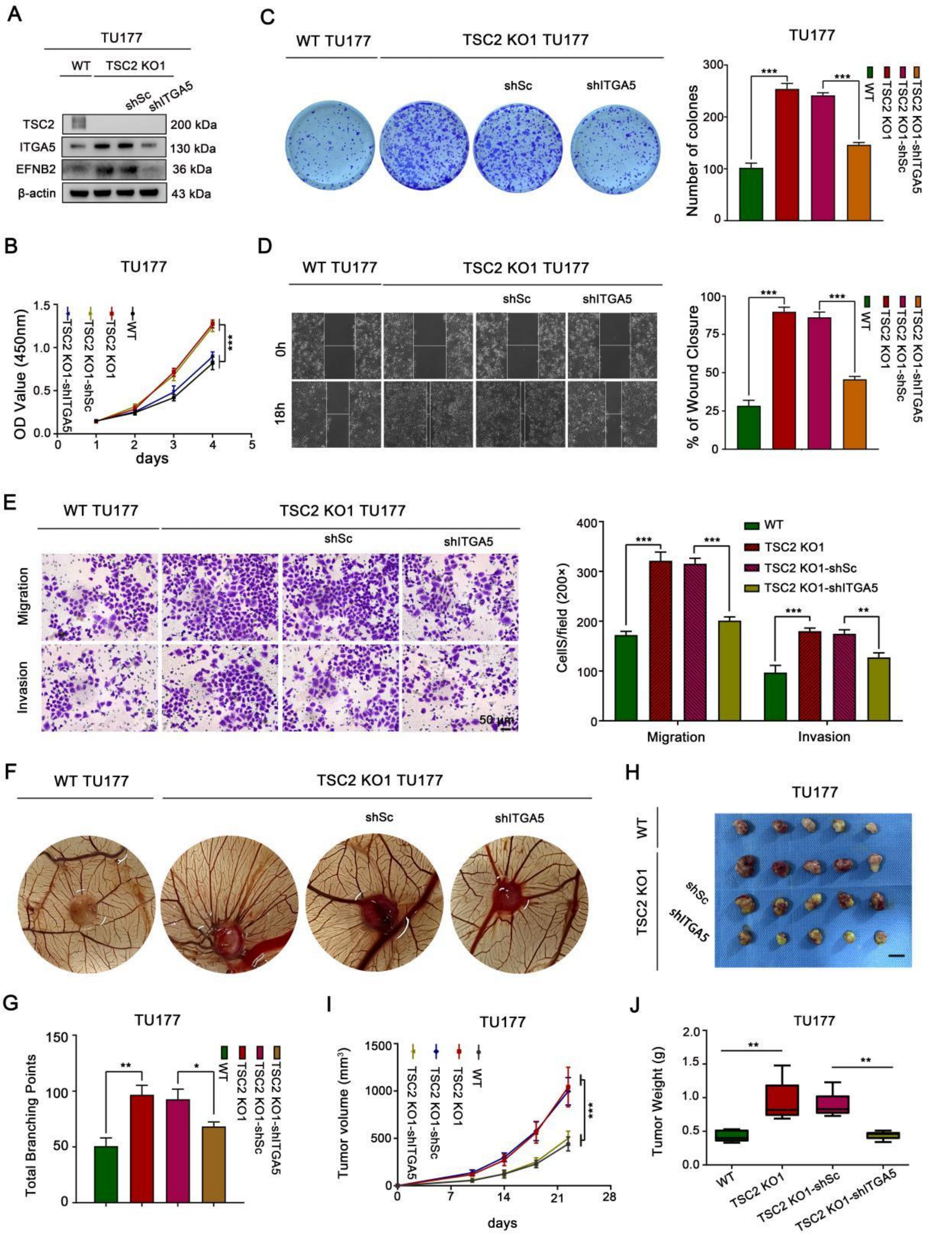

Figure S4. Depletion of ITGA5 reduced the tumor-promoting effect of TSC2 knockout on the TU177 cells. (A-J) TSC2 KO1 TU177 cells were infected with ITGA5 shRNAs-expressing (shITGA5) lentiviruses or shSc. Cell lysates of the indicated cells were subjected to western blotting **(A)**. Cell growth rates, migration

and invasion abilities of the indicated cells were evaluated by CCK-8 assays **(B)**, colony formation assays **(C)**, wound healing assays **(D)** and transwell assays **(E)**, (**C-E, left panel**: representative images; **right panel**: statistical analysis). Scale bars, 50 μm. Data were indicated as mean ± SD of triplicate technical replicates. **P < 0.01, ***P < 0.001. The indicated cells were subjected to CAM assays **(F-G)**, representative images **(F)** and statistical analysis **(G)** are shown. The error bars represent mean ± SD (n = 6 per group). *P < 0.05; **P < 0.01. **(H-J)** The indicated cells were subcutaneously injected into mice for monitoring tumor growth. The tumor images **(H)**, tumor volumes **(I)** and tumor weights **(J)** were shown. Error bars indicate mean ± SD (n = 5 mice/group). **P < 0.01, ***P < 0.001. Scale bars, 1 cm **(H)**.

Figure S5

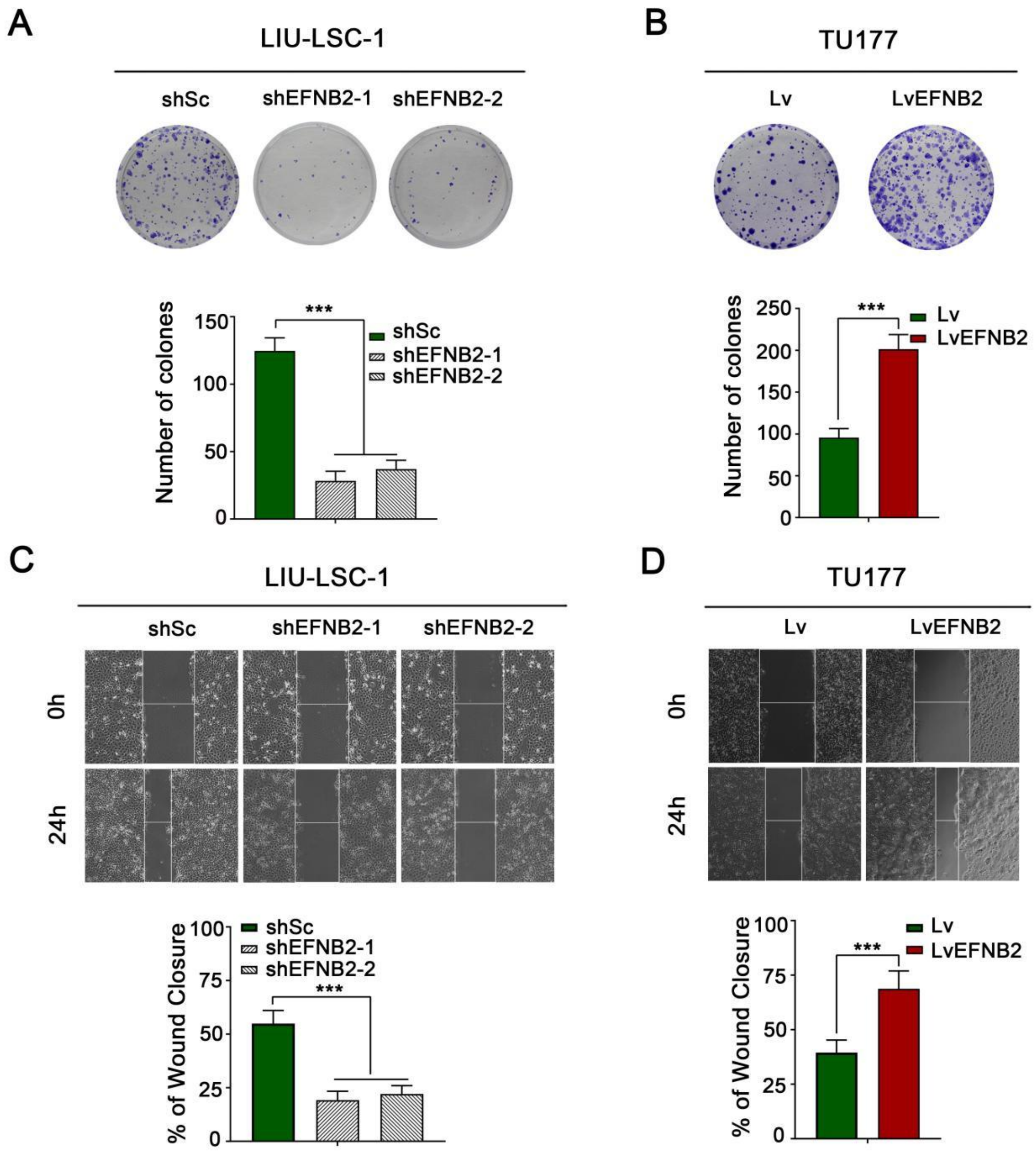

Figure S5. EFNB2 promotes LSCC cells proliferation and migration. (A, C) EFNB2 shRNA-expressing (shEFNB2-1 or shEFNB2-2) LIU-LSC-1 cells and their control cells (shSc). **(B, D)** EFNB2-overexpressing (LvEFNB2) TU177 cells and their counterpart control cells (Lv). Cell proliferation was determined by colony formation assay **(A-B)**. Cell migration was assessed by wound healing assay **(C-D)**. The error bars represent the mean ± SD of triplicate independent experiments. ***P < 0.001.

Figure S6

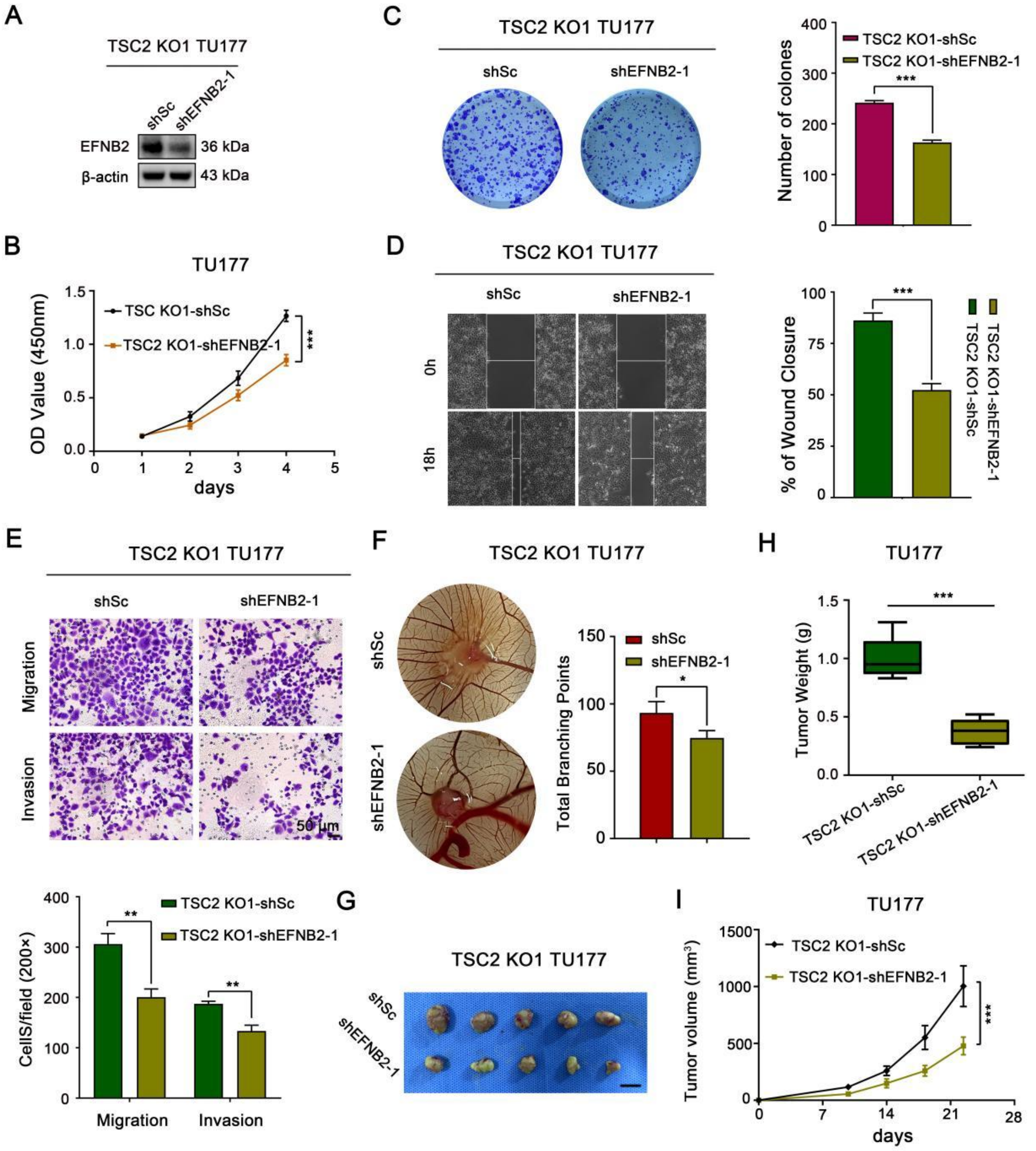

Figure S6. Silencing of EFNB2 reduced the proliferation, migration, invasion, angiogenesis and tumor growth abilities of the TSC2 KO1 TU177 cells. (A-I) The TSC2 KO1 TU177 cells were infected with EFNB2 shRNAs-expressing (shEFNB2-1) or control shRNA- (shSc) lentiviruses. The EFNB2 expression was assessed using western blotting **(A)**. Cell growth rates, migration and invasion abilities of the indicated cells were evaluated by CCK-8 assays **(B)**, colony formation assays **(C, left**

panel: representative images; **right panel**: statistical analysis), wound healing assays (**D, left panel**: representative images; **right panel**: statistical analysis) and transwell assays (**E, upper panel**: representative images; **lower panel**: statistical analysis). Scale bars, 50 μm. Data were indicated as mean ± SD of triplicate technical replicates. $^{**}P < 0.01$, $^{***}P < 0.001$. The indicated cells were subjected to CAM assays (**F, left panel**: representative images; **right panel**: statistical analysis). The error bars represent mean ± SD (n = 6 per group). $^{*}P < 0.05$. **(G-I)** The indicated cells were subcutaneously injected into mice for monitoring tumor growth. The tumor images **(G)** were recorded. The tumor weights **(H)** and tumor volumes **(I)** were quantified. Error bars indicate mean ± SD (n = 5 mice/group). $^{***}P < 0.001$. Scale bars, 1 cm **(G)**.

Figure S7

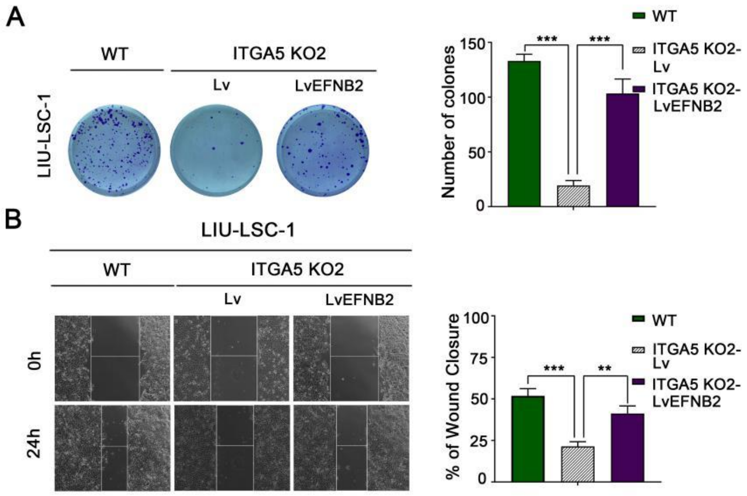

Figure S7. The reduction of proliferation and migration caused by ITGA5 knockout were partly rescued by ectopic expression of EFNB2 in LIU-LSC-1 cells. (A-B) Proliferative and migratory abilities of EFNB2-overexpressing ITGA5 KO LIU-LSC1 cells, empty vector-expressing ITGA5 KO LIU-LSC-1 cells and ITGA5 WT LIU-LSC-1 cells were measured by colony formation **(A)** and wound healing assays **(B)**, respectively. The error bars represent the mean ± SD of triplicate independent experiments. **P < 0.01; ***P < 0.001.

Figure S8

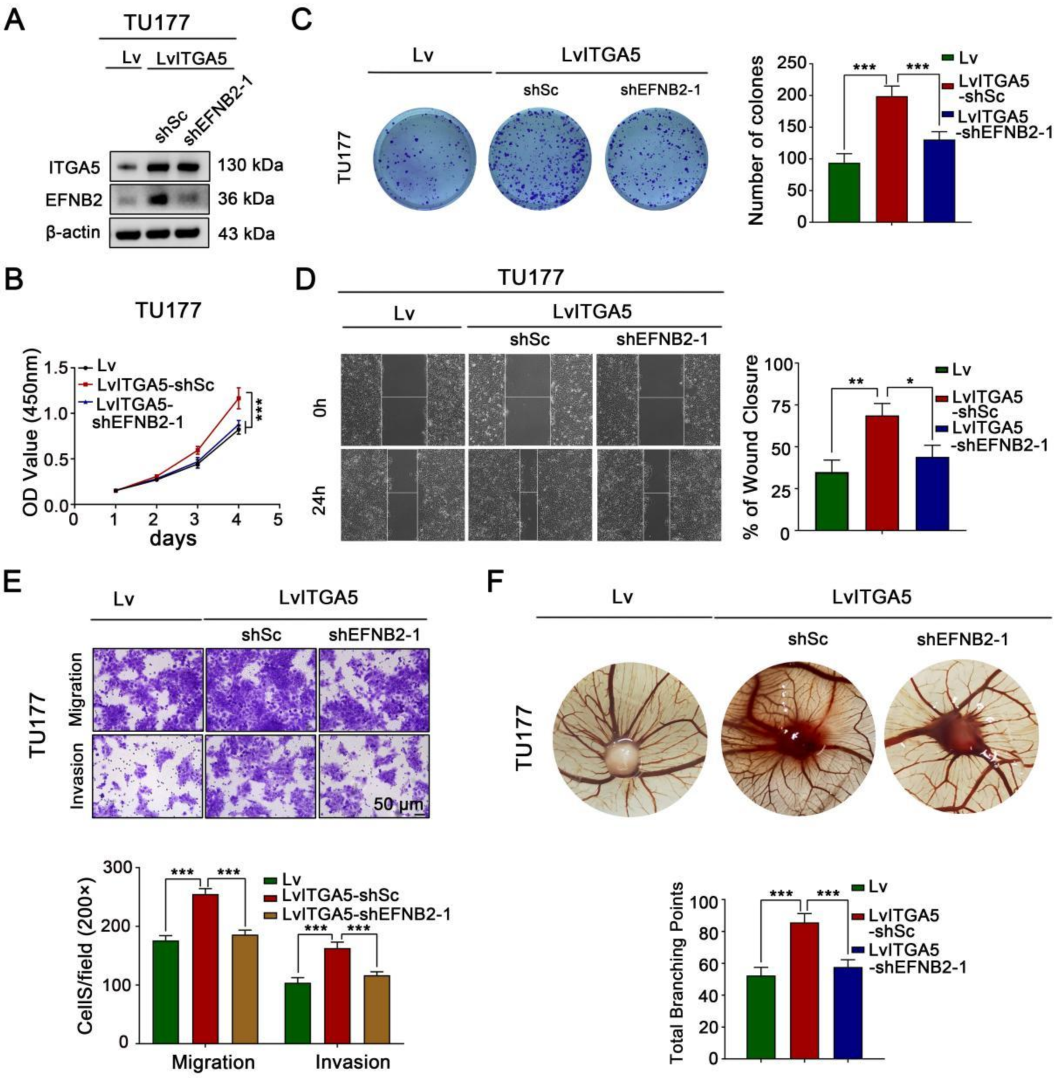

Figure S8. The enhancement of LSCC tumor progression mediated by ITGA5 overexpression was attenuated by knockdown of EFNB2. (A-F) EFNB2 shRNA- (shEFNB2-1) or control shRNA- (shSc) expressing lentiviruses were transduced to ITGA5-overexpression TU177 cells and control cells. The protein expression of ITGA5 and EFNB2 were detected by western blotting **(A)**. The cells were subjected to CCK-8 **(B)**, colony formation **(C)**, wound healing **(D)**, transwell **(E)** and the CAM assays **(F)**. **(B-E)** The error bars represent the mean ± SD of triplicate independent

experiments. *P < 0.05; **P < 0.01; ***P < 0.001. Scale Bars, 50 μm. **(F)** Error bars represent mean ± SD (n = 6 per group). **P < 0.01; ***P < 0.001.

Figure S9

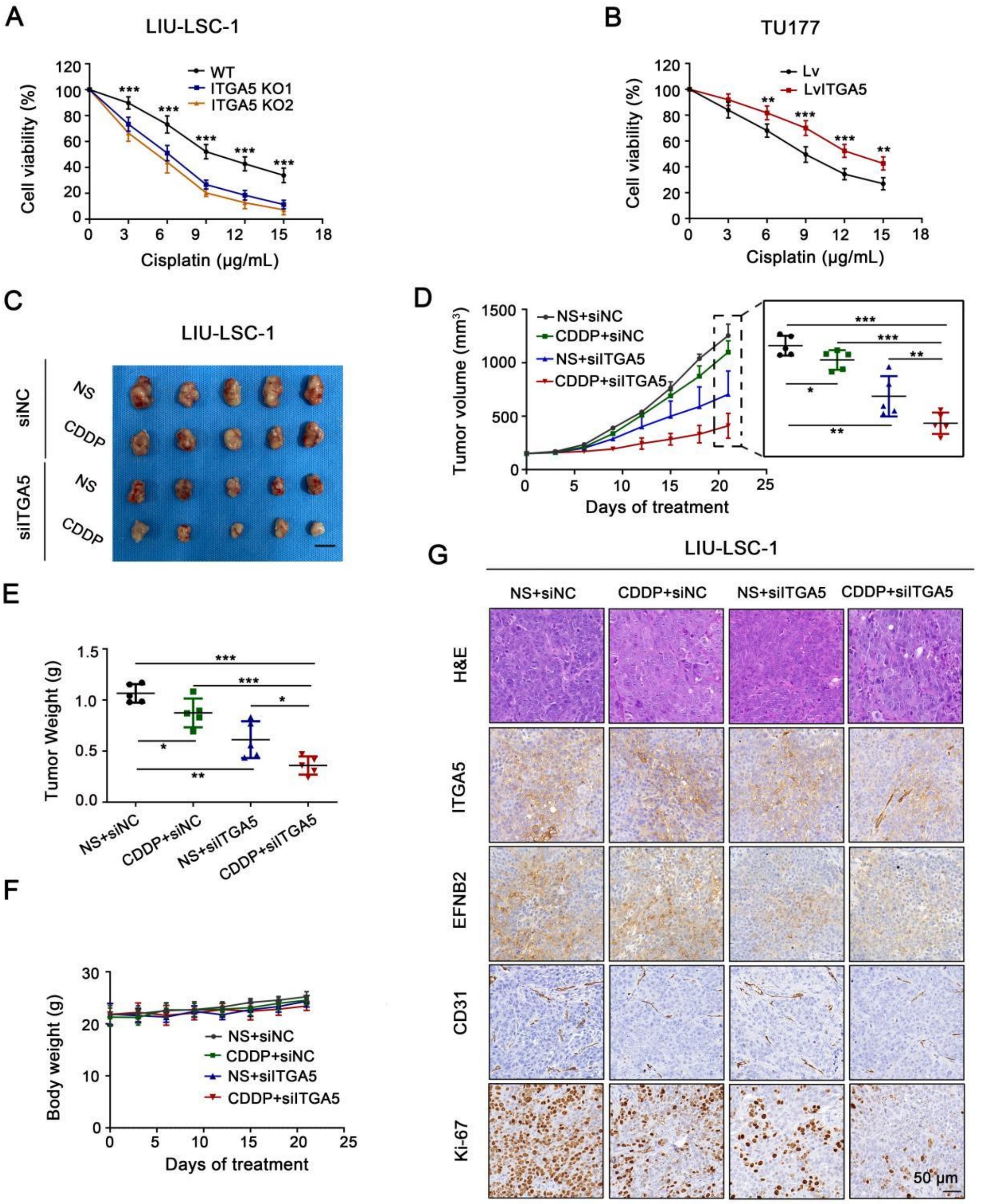

Figure S9. Depletion of ITGA5 increases chemosensitivity of CDDP in LSCC cells. (A-B) The indicated cells were treated with various concentration of CDDP for 24 h, and cell viability was detected with CCK-8 assay. The error bars represent the mean ± SD of triplicate technical replicates. ***P < 0.001. **(C-G)** Effects of CDDP combined with ITGA5 siRNAs on LIU-LSC-1 xenograft tumor growth. Tumor

images **(C)**, tumor volume **(D)**, tumor weight **(E)**, and body weight of mice **(F)** were displayed. Scale bar, 1 cm. Representative IHC images of ITGA5, EFNB2, CD31 and Ki-67 in subcutaneous xenografts **(G)**. Scale bar, 50 μm. Error bars indicate mean ± SD (n = 5 mice/group). *P < 0.05; **P < 0.01; ***P < 0.001.

Figure S10

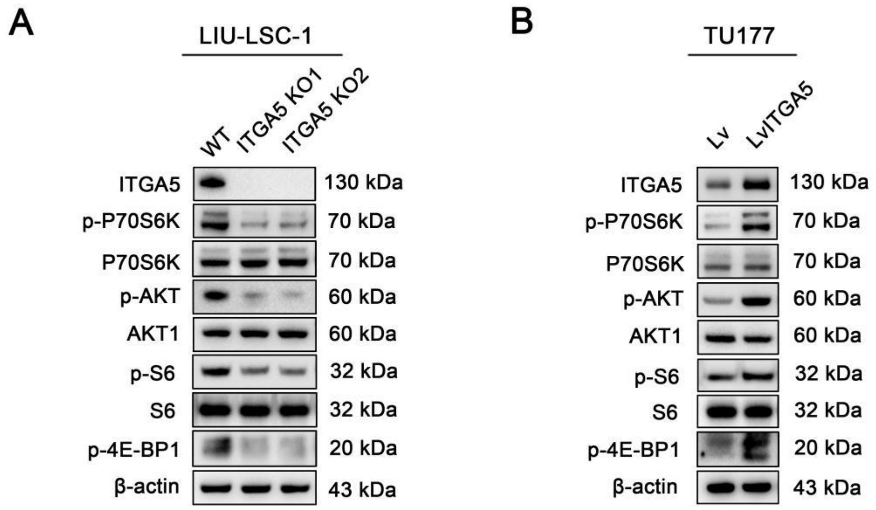

Figure S10. ITGA5 positively regulates the activity of AKT and mTORC1 in LSCC cells. (A-B) WT and ITGA5 KO LIU-LSC-1 cells **(A)**; ITGA5-overexpressed TU177 cells (LvITGA5) and control cells (Lv) **(B)**. Cell lysates were subjected to western blotting with the indicated antibodies **(A-B)**.

Figure S11

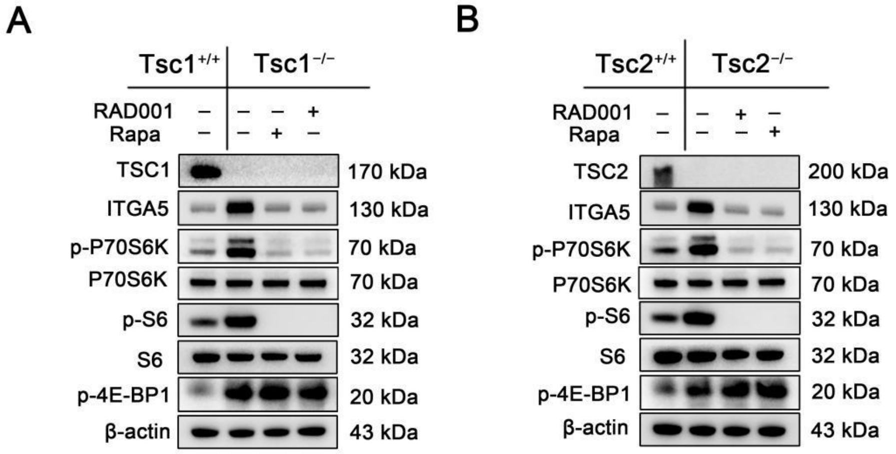

Figure S11. mTORC1 enhances ITGA5 expression in Tsc1$^{-/-}$ or Tsc2$^{-/-}$ MEFs. **(A-B)** Tsc1$^{-/-}$ **(A)** and Tsc2$^{-/-}$ MEFs **(B)** were treated with mTORC1 inhibitors (20 nM Rapa or 50 nM RAD001) for 24 h. Cell lysates were subjected to western blot analysis.

Figure S12

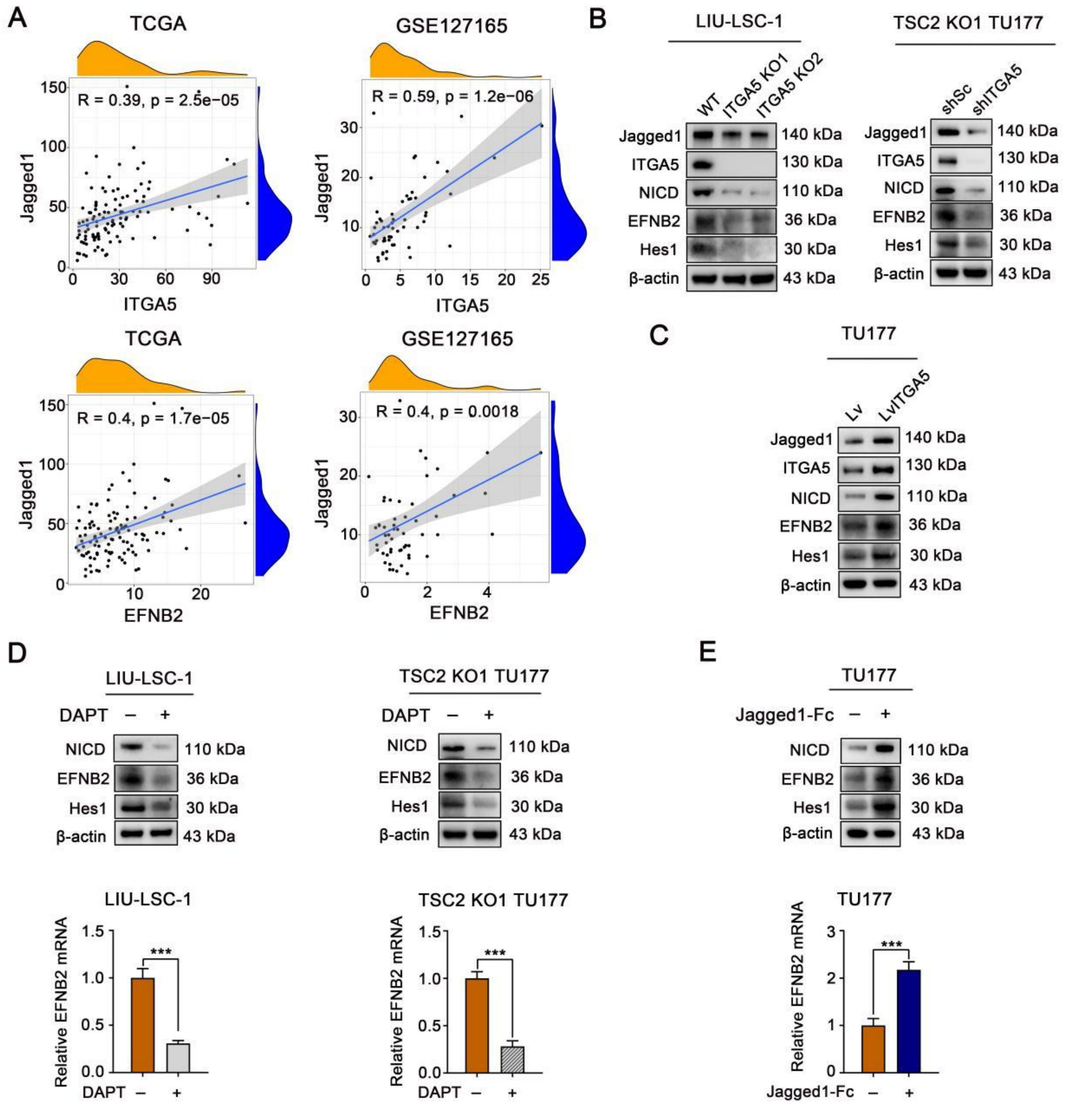

Figure S12. ITGA5 upregulates the expression of EFNB2 through the activation of the Jagged1/Notch1 pathway. (A) The correlation between Jagged1 and ITGA5 or EFNB2 expression was analyzed by a Pearson's correlation analysis using the TCGA and GSE127165 cohorts. **(B-C)** Cell lysates of the indicated genetically engineered LSCC cells were subjected to western blotting with the indicated antibodies. **(D-E)** The LIU-LSC-1 and TSC2 KO1 TU177 cells were treated with 10 μM DAPT for 24 h (D); the TU177 cells were treated with 1μg/mL Jagged1-Fc for 24

h **(E)**. The samples were subjected to western blotting **(upper panel of D and E)** and qRT-PCR **(lower panel of D and E)** analyses, respectively. The error bars represent the mean ± SD of triplicate technical replicates. ***P < 0.001.

SUPPLEMENTARY TABLES

Table S1. Clinical features of 94 LSCC patients.

Parameters	Number of Cases（%）
Age	
< 60	34（36.2）
≥ 60	60（63.8）
Gender	
Female	9（9.6）
Male	85（90.4）
T Stage[1]	
T1	14（14.9）
T2	22（23.4）
T3	27（28.7）
T4	31（33.0）
lymph node metastasis	
N0	61（64.9）
N+	33（35.1）
Distant metastasis	
M0	91（96.8）
M1	3（3.2）

[1]TNM Staging is referring to the AJCC 8th edition TNM Staging Criteria.

Table S2. Correlation between p-S6 expression and clinicopathological characteristics of LSCC patients.

Characteristic	p-S6 expression		p-value	Method
	High	Low		
n	47	47		
Age, mean ± SD	62.28 ± 12.04	62.21 ± 10.08	0.978	T test
Gender, n (%)			1.000	Fisher.test
Female	5 (5.3%)	4 (4.3%)		
Male	42 (44.7%)	43 (45.7%)		
T Stage, n (%)			< 0.001	Chisq.test
T1	4 (4.3%)	10 (10.6%)		
T2	5 (5.3%)	17 (18.1%)		
T3	13 (13.8%)	14 (14.9%)		
T4	25 (26.6%)	6 (6.4%)		
Lymph node metastasis, n (%)			0.006	Fisher.test
N0	23 (24.5%)	38 (40.4%)		
N1	5 (5.3%)	3 (3.2%)		
N2	14 (14.9%)	3 (3.2%)		
N3	5 (5.3%)	3 (3.2%)		
Distant metastasis, n (%)			1.000	Fisher.test
M0	46 (48.9%)	45 (47.9%)		
M1	1 (1.1%)	2 (2.1%)		

Table S3. Correlation between ITGA5 expression and clinicopathological characteristics of LSCC patients.

Characteristic	ITGA5 expression		p-value	Method
	High	Low		
n	47	47		
Age, mean ± SD	62.49 ± 12.18	62 ± 9.91	0.831	T test
Gender, n (%)			1.000	Fisher.test
Female	4 (4.3%)	5 (5.3%)		
Male	43 (45.7%)	42 (44.7%)		
T Stage, n (%)			< 0.001	Chisq.test
T1	4 (4.3%)	10 (10.6%)		
T2	4 (4.3%)	18 (19.1%)		
T3	12 (12.8%)	15 (16%)		
T4	27 (28.7%)	4 (4.3%)		
Lymph node metastasis, n (%)			< 0.001	Fisher.test
N0	21 (22.3%)	40 (42.6%)		
N1	5 (5.3%)	3 (3.2%)		
N2	14 (14.9%)	3 (3.2%)		
N3	7 (7.4%)	1 (1.1%)		
Distant metastasis, n (%)			1.000	Fisher.test
M0	45 (47.9%)	46 (48.9%)		
M1	2 (2.1%)	1 (1.1%)		

Table S4. Correlation between EFNB2 expression and clinicopathological characteristics of LSCC patients.

Characteristic	EFNB2 expression		p	Method
	High	Low		
n	47	47		
Age, mean ± SD	61.64 ± 11.01	62.85 ± 11.17	0.597	T test
Gender, n (%)			1.000	Fisher.test
Female	5 (5.3%)	4 (4.3%)		
Male	42 (44.7%)	43 (45.7%)		
T Stage, n (%)			< 0.001	Chisq.test
T1	7 (7.4%)	7 (7.4%)		
T2	6 (6.4%)	16 (17%)		
T3	9 (9.6%)	18 (19.1%)		
T4	25 (26.6%)	6 (6.4%)		
Lymph node metastasis, n (%)			0.025	Fisher.test
N0	24 (25.5%)	37 (39.4%)		
N1	4 (4.3%)	4 (4.3%)		
N2	13 (13.8%)	4 (4.3%)		
N3	6 (6.4%)	2 (2.1%)		
Distant metastasis, n (%)			1.000	Fisher.test
M0	45 (47.9%)	46 (48.9%)		
M1	2 (2.1%)	1 (1.1%)		

Table S5. STR analysis of LIU-LSC-1 cell line.

	LIU-LSC-1 cell line	
STR alleles	Allele1	Allele2
D5S818	12	
D13S317	8	
D7S820	8	11
D16S539	11	
VWA	16	
TH01	8	
AMEL	X	Y
TPOX	8	11
CSF1PO	10	12
D12S391	15	22
FGA	23	
D2S1338	21	23
D21S11	29	
D18S51	20	
D8S1179	13	14
D3S1358	16	
D6S1043	11	12
PENTAE	16	22
D19S433	15.2	16.2
PENTAD	13	

Table S6. Cell lines and growth medium.

Cell line	Source	Tissue source	Complete growth medium
TU177	Otwo Biotech Inc. (Shenzhen, China).	laryngeal SCC	RPMI 1640 (Gibco: Cat# 11875093) + 10% FBS (Biological Industries, Cat# 04-001-1ACS) + 1% penicillin/streptomycin (Beyotime, Cat# C0222)
LIU-LSC-1	newly established cell line	laryngeal SCC	Epithelial Cell Complete Medium (VivaCell: Cat# C3660-0100) + 1% penicillin/streptomycin (Beyotime, Cat# C0222)
AMC-HN-8	Otwo Biotech Inc. (Shenzhen, China).	laryngeal SCC	RPMI 1640 (Gibco: Cat# 11875093) + 10% FBS (Biological Industries, Cat# 04-001-1ACS + 1% penicillin/streptomycin (Beyotime, Cat# C0222)
Tsc1$^{+/+}$	described previously	Murine embryonic fibroblasts (MEFs)	DMEM (Gibco: Cat# 11995065) + 10% FBS (Biological Industries, Cat# 04-001-1ACS + 1% penicillin/streptomycin (Beyotime, Cat# C0222)
Tsc1$^{-/-}$	described previously	Murine embryonic fibroblasts (MEFs)	DMEM (Gibco: Cat# 11995065) + 10% FBS (Biological Industries, Cat# 04-001-1ACS + 1% penicillin/streptomycin (Beyotime, Cat# C0222)
Tsc2$^{+/+}$	described previously	Murine embryonic fibroblasts (MEFs)	DMEM (Gibco: Cat# 11995065) + 10% FBS (Biological Industries, Cat# 04-001-1ACS + 1% penicillin/streptomycin (Beyotime, Cat# C0222)
Tsc2$^{-/-}$	described previously	Murine embryonic fibroblasts (MEFs)	DMEM (Gibco: Cat# 11995065) + 10% FBS (Biological Industries, Cat# 04-001-1ACS + 1% penicillin/streptomycin (Beyotime, Cat# C0222)
HEK293T	ATCC（Manassas,	kidney; Embryo	DMEM (Gibco: Cat#

VA,USA）	11995065) + 10% FBS (Biological Industries, Cat# 04-001-1ACS + 1% penicillin/streptomycin (Beyotime, Cat# C0222)

Table S7. Detailed information of antibodies.

ANDIBODYS	SOURCE	IDENTIFIER
Mouse monoclonal antibody anti-Ki-67	Cell Signaling Technology	Cat# 9027S
Mouse monoclonal antibody anti-beta-Actin	Sigma-Aldrich	Cat# A1978
Rabbit monoclonal antibody anti-ITGA5	Abcam	Cat# ab150361
Rabbit monoclonal antibody anti-EFNB2	Sigma-Aldrich	Cat# HPA008999
Rabbit monoclonal antibody anti-HIF-1α	Abcam	Cat# ab228649
Rabbit monoclonal antibody anti-p-S6 (S235/236)	Cell Signaling Technology	Cat# 4857S
Rabbit monoclonal antibody anti-S6	Cell Signaling Technology	Cat# 2217S
Rabbit monoclonal antibody anti-mTOR	Cell Signaling Technology	Cat# 2983P
Rabbit monoclonal antibody anti-Rictor	Cell Signaling Technology	Cat# 2114S
Rabbit monoclonal antibody anti-Raptor	Cell Signaling Technology	Cat# 2280S
Rabbit monoclonal antibody anti-CD31	Abcam	Cat# ab76533
Goat Anti-Rabbit IgG H&L (FITC)	Abcam	Cat# ab6717
Goat Anti-Mouse IgG HRP	Abcam	Cat# ab6789
Goat Anti-Rabbit IgG HRP	Abcam	Cat# ab6721
Mouse monoclonal antibody anti-CD44	Abcam	Cat# ab6124
Rabbit monoclonal antibody anti- Jagged1	Abcam	Cat# ab109536
Rabbit monoclonal antibody anti-p-mTOR(S2448)	Cell Signaling Technology	Cat# 5536S
Rabbit monoclonal antibody anti-TSC1	Cell Signaling Technology	Cat# 6935S
Rabbit monoclonal antibody anti-TSC2	Cell Signaling Technology	Cat# 4308S
Rabbit monoclonal antibody anti-p-AKT(S473)	Cell Signaling Technology	Cat# 4060S

Rabbit monoclonal antibody anti-AKT1	Cell Signaling Technology	Cat# 75692S
Rabbit monoclonal antibody anti-NICD	Cell Signaling Technology	Cat# 4147S
Rabbit monoclonal antibody anti-p-4E-BP1 (T37/46)	Cell Signaling Technology	Cat# 2855S
Rabbit monoclonal antibody anti-Hes1	Abcam	Cat#ab108937
Rabbit polyclonal antibody anti-P70S6K (phospho T389)	Abcam	Cat# ab2571
Rabbit monoclonal antibody anti-P70S6K	Abcam	Cat# ab32529

Table S8. Primer information for qRT-PCR analysis.

Primer name	GC content (%)	Tm (°C)	Annealing Temperature (°C)	Primer sequence
β-actin FORWARD	52.2	58.5	60	CTG GCA CCA CAC CTT CTA CAA TG
β-actin REVERSE	61.9	61.3		GGC GTA CAG GGA TAG CAC AGC
ITGA5 FORWARD	45.5	54.6	60	CAT GAT GAG TTT GGC CGA TTT G
ITGA5 REVERSE	45.5	54.2		CCC CCA GGA AAT ACA AAC ACT A
EFNB2 FORWARD	40.9	53.2	60	TAA AGA TCC AAC AAG ACG TCC A
EFNB2 REVERSE	45.5	53.3		CGT GAT GAT GAT GAC GAT GAA G
RAPTOR FORWARD	50.0	56.0	60	GAC ACG GAA GAT GTT CGA CAA G
RAPTOR REVERSE	50.0	54.7		ATC TGA GAA GCA ACG CTC TC

Table S9. Differentially expressed genes of the RNA-seq (shRaptor-1 LIU-LSC-1 cells vs. shSc LIU-LSC-1 cells).

Table S10. The top 10 enriched pathways of down-regulated differentially expressed genes in shRaptor-1 LIU-LSC-1 cells compared to the control cells (shSc LIU-LSC-1 cells).

Table S11. Differentially expressed genes of the RNA-seq (ITGA5 KO2 LIU-LSC-1 cells vs. WT LIU-LSC-1 cells).
